# External Evaluation of Risperidone Population Pharmacokinetic Models Using Opportunistic Pediatric Data

**DOI:** 10.3389/fphar.2022.817276

**Published:** 2022-03-17

**Authors:** Eleni Karatza, Samit Ganguly, Chi D. Hornik, William J. Muller, Amira Al-Uzri, Laura James, Stephen J. Balevic, Daniel Gonzalez

**Affiliations:** ^1^ Division of Pharmacotherapy and Experimental Therapeutics, UNC Eshelman School of Pharmacy, The University of North Carolina at Chapel Hill, Chapel Hill, NC, United States; ^2^ Regeneron Pharmaceuticals, Inc., Tarrytown, NY, United States; ^3^ Duke Clinical Research Institute, Durham, NC, United States; ^4^ Ann and Robert H. Lurie Children’s Hospital of Chicago, Chicago, IL, United States; ^5^ Oregon Health and Science University, Portland, OR, United States; ^6^ Arkansas Children’s Hospital Research Institute and the University of Arkansas for Medical Sciences, Little Rock, AR, United States

**Keywords:** risperidone, pediatrics, pharmacokinetics, precision dosing, population modeling

## Abstract

Risperidone is approved to treat schizophrenia in adolescents and autistic disorder and bipolar mania in children and adolescents. It is also used off-label in younger children for various psychiatric disorders. Several population pharmacokinetic models of risperidone and 9-OH-risperidone have been published. The objectives of this study were to assess whether opportunistically collected pediatric data can be used to evaluate risperidone population pharmacokinetic models externally and to identify a robust model for precision dosing in children. A total of 103 concentrations of risperidone and 112 concentrations of 9-OH-risperidone, collected from 62 pediatric patients (0.16–16.8 years of age), were used in the present study. The predictive performance of five published population pharmacokinetic models (four joint parent-metabolite models and one parent only) was assessed for accuracy and precision of the predictions using statistical criteria, goodness of fit plots, prediction-corrected visual predictive checks (pcVPCs), and normalized prediction distribution errors (NPDEs). The tested models produced similarly precise predictions (Root Mean Square Error [RMSE]) ranging from 0.021 to 0.027 nmol/ml for risperidone and 0.053–0.065 nmol/ml for 9-OH-risperidone). However, one of the models (a one-compartment mixture model with clearance estimated for three subpopulations) developed with a rich dataset presented fewer biases (Mean Percent Error [MPE, %] of 1.0% *vs*. 101.4, 146.9, 260.4, and 292.4%) for risperidone. In contrast, a model developed with fewer data and a more similar population to the one used for the external evaluation presented fewer biases for 9-OH-risperidone (MPE: 17% *vs*. 69.9, 47.8, and 82.9%). None of the models evaluated seemed to be generalizable to the population used in this analysis. All the models had a modest predictive performance, potentially suggesting that sources of inter-individual variability were not entirely captured and that opportunistic data from a highly heterogeneous population are likely not the most appropriate data to evaluate risperidone models externally.

## Introduction

Risperidone is the most frequently prescribed atypical antipsychotic in the pediatric population (Halfdanarson et al., 2017). It is an antagonist of serotoninergic, dopaminergic, adrenergic, and histaminergic receptors (Chopko et al., 2018). In the United States, risperidone is indicated for use in the pediatric population for the treatment of irritability associated with autistic disorder (5–16 years of age), bipolar disorder (10–17 years of age), and schizophrenia (13–17 years of age) (Risperdal^®^ package insert, 2009). In addition, risperidone is frequently used off-label (including in pediatric patients below 2 years of age) for the management of delirium in the pediatric intensive care unit (PICU), and in children greater than 5 years of age to treat post-traumatic stress disorder, Tourette syndrome, and agitation associated with delirium (Campbell et al., 2020; Liviskie and McPherson, 2021). Risperidone has also been demonstrated to be an efficacious option for the management of attention-deficit/hyperactivity disorder and various other psychiatric disorders associated with anxiety and irritability in children (Eapen and Gururaj, 2005; Jensen et al., 2007; Biederman et al., 2008; Arnold et al., 2015; Lee et al., 2018). Risperidone use is associated with dose and duration-dependent adverse effects, including weight gain, extrapyramidal symptoms, prolactin elevation, sedation, and QTc interval prolongation (Vanwong et al., 2020; Kloosterboer et al., 2021).

Risperidone is extensively metabolized by cytochrome P450 (CYP) 2D6 and 3A4, leading to the formation of its active metabolite 9-OH-risperidone. Most of the drug is excreted as metabolites in the urine (65%) and the feces (14%), while only 5% is recovered unchanged in the urine (Sheehan et al., 2010; Saibi et al., 2012; Kneller et al., 2020; Kneller and Hempel, 2020). There is large inter-and intra- individual variability in risperidone’s plasma concentrations, which has been attributed primarily to genetic polymorphisms in *CYP2D6* and secondarily to age, renal and hepatic function, disease status, and comedications (Livingston, 1994; Sheehan et al., 2010; Saibi et al., 2012; Mauri et al., 2018; Kneller et al., 2020; Kneller and Hempel, 2020). Despite its wide usage, a therapeutic window has not yet been established. Only recently, a range of 15–25 μg/L plasma concentrations, has been proposed as a plausible therapeutic window for the treatment of ADHD of a 10-year-old child receiving risperidone for over 3 months without comedications (Kloosterboer et al., 2021). Risperidone’s large variability in plasma concentrations is anticipated to influence its efficacy and toxicity profile. Population pharmacokinetic (PK) models may offer an approach to identify sources of inter-individual variability and to inform precision dosing that would support efficacy for the use of risperidone in children (Medhasi et al., 2016; Kloosterboer et al., 2021).

A model with acceptable predictive performance is needed to guide precision dosing. The vast majority of population PK models of risperidone and 9-OH-risperidone have been developed using data from adult populations (Vermeulen et al., 2007; Feng et al., 2008; Locatelli et al., 2010; Yoo et al., 2012; Vandenberghe et al., 2015; Ji et al., 2016), while only three pediatric population PK models have been developed (Thyssen et al., 2010; Sherwin et al., 2012; Kloosterboer et al., 2021). In most cases, the disposition of both risperidone and its active metabolite was characterized with a one-compartment model. To account for the high variability in risperidone concentrations, mixture models were applied to estimate clearance for multiple subpopulations or *CYP2D6* genotype was included in the model as a covariate (Locatelli et al., 2010; Yoo et al., 2012; Vandenberghe et al., 2015). Other covariates that were identified to impact risperidone or 9-OH-risperidone PK were age and weight. More specifically, 9-OH-risperidone’s clearance was shown to decrease with increasing age in adult populations (Feng et al., 2008; Vandenberghe et al., 2015). Also, weight was used for allometric scaling of the clearance and volume of distribution in all the models developed with pediatric data, accounting for changes in body size (Thyssen et al., 2010; Sherwin et al., 2012; Kloosterboer et al., 2021).

Models intended for precision dosing should undergo an extensive internal and external evaluation to ensure their reliability for drug dosing optimization. The most stringent method to effectively assess the predictive performance and generalizability of a population PK model in other populations is the external evaluation (Hwang et al., 2017; US FDA, 2019; Cheng et al., 2021). However, in most cases, only an internal evaluation is carried out during population PK model development (Hwang et al., 2017; Wu et al., 2021). Only one of the population PK models developed for risperidone and 9-OH-risperidone has been externally evaluated, using PK data collected in adults (Ji et al., 2016).

Opportunistic data (i.e., data collected during routine clinical care without retrieving samples solely for research purposes) have helped develop population PK models to support dosing selection in pediatrics (Gonzalez et al., 2014; Ge et al., 2020). The present study aimed to assess if sparse opportunistic data from a highly heterogeneous pediatric population can be used to perform an external evaluation of published models for risperidone. Secondarily, this study aimed to explore which of the published models of risperidone and 9-OH-risperidone is more generalizable to other populations and thus can be used for precision dosing.

## Materials and Methods

### Data Collection

The plasma concentrations of risperidone and 9-OH-risperidone used for the present external evaluation analysis were collected through the Pediatric Trials Network (PTN) Pharmacokinetics of Understudied Drugs Administered to Children Per Standard of Care trial (POPS; Clinical Trials. gov # NCT01431326). POPS is a multicenter, prospective study of the PK of understudied drugs, including risperidone, administered to children (<21 years of age) per standard of care, as administered by their treating caregiver. The study protocol was reviewed and approved by the institutional review boards of Duke University (coordinating center) and all participating study sites. All participants and participant parents/legal guardians provided written informed consent or assent, as applicable. Exclusion criteria included known pregnancy, as determined by interview or testing, if available.

Depending on the patient’s age and clinical condition, risperidone was administered through various routes, namely oral, nasogastric/orogastric, nasojejunal, transpyloric, gastrostomy tube, and jejunostomy tube. In addition, different formulations, such as solution and tablet, were used.

Blood samples were collected in ethylenediaminetetraacetic acid (EDTA) containing tubes during clinical laboratory collections or following a specific collection for study purposes. Plasma was separated by centrifugation (2,000 g) for 10 min at 4°C and stored at −70°C or colder within 8 h of collection. Given that this was a standard of care study, dosing and sampling schemes varied between subjects. In the study protocol, recommended PK sampling windows were provided, but PK samples collected with a standard of care laboratory assessment were also acceptable. Standard of care laboratory assessments (e.g., comprehensive metabolic panel) were recorded if collected within 72 h of a study dose of the drug.

### Analytical Method

Plasma samples were analyzed using a validated liquid chromatography method with tandem mass spectrometric detection (LC-MS/MS) by Frontage Laboratories (Exton, PA). Risperidone and its metabolite were extracted by protein precipitation using acetonitrile. Reversed-phase high-performance liquid chromatography (HPLC) separation was achieved using a Phenomenex Kinetex ^®^ PFP column (50 × 3 mm, 2.6 micron). A gradient of two mobile phases was used with phase A consisting of 5 mM ammonium formate and 0.02% formic acid in water and acetonitrile 50/50 v/v and phase B consisting of 5 mM ammonium formate and 0.02% formic acid in water and acetonitrile 2/98 v/v. MS/MS detection was set at mass transitions of m/z 411.2→191.2 for risperidone and m/z 427.2 → 207.2 for 9-OH-risperidone. The lower limit of quantitation (LLOQ) for risperidone and 9-OH-risperidone was 0.100 ng/ml. The linear range of the method was 0.100–100 ng/ml for both compounds. Sample freeze-thaw stability was demonstrated for three cycles (freeze at −70°C and thaw to room temperature).

### Models Under Evaluation

A literature search was performed in PubMed using search terms as “risperidone,” “pharmacokinetics,” and “population model.” Inclusion criteria applied for selecting studies for the external evaluation analysis were studies where risperidone was administered orally and studies with relatively large sample sizes (at least 40 patients included). The published results of the model’s internal evaluation were considered.

A total of nine population PK models were identified to meet the above criteria (Vermeulen et al., 2007; Feng et al., 2008; Locatelli et al., 2010; Thyssen et al., 2010; Sherwin et al., 2012; Yoo et al., 2012; Vandenberghe et al., 2015; Ji et al., 2016; Kloosterboer et al., 2021). Three models used pediatric data (Thyssen et al., 2010; Sherwin et al., 2012; Kloosterboer et al., 2021), while the rest were developed with data from adults (Vermeulen et al., 2007; Feng et al., 2008; Locatelli et al., 2010; Yoo et al., 2012; Vandenberghe et al., 2015; Ji et al., 2016). As *CYP2D6* genotyping data were not available in our dataset (POPS study), for this external evaluation analysis, three models that included *CYP2D6* genotype as a covariate were excluded (Locatelli et al., 2010; Yoo et al., 2012; Vandenberghe et al., 2015). Similarly, one study where co-administration of carbamazepine was found as a covariate significantly altering clearance was excluded (Vermeulen et al., 2007).

As the study’s primary aim was to evaluate population PK models developed in pediatric populations externally, all the models developed with pediatric data (Thyssen et al., 2010; Sherwin et al., 2012; Kloosterboer et al., 2021) were included. The only model developed using solely adult data that was included in the present analysis was the model developed by Feng et al., 2008. This model was included as it was developed with the largest number of observations for both compounds (1,236 concentrations of risperidone and 1,236 concentrations of 9-OH risperidone) obtained from a large (490 patients) and highly heterogeneous population (18–93 years old and 42–187 kg of weight). In addition, the model developed by Feng et al., 2008 was used as a basis by Sherwin et al., 2012 to develop a model using only data from a pediartic population. More precisely, Sherwin et al., 2012 used the same structure and number of parameters as the model developed by Feng et al., 2008. A summary of the models included in the external evaluation analyses is provided in Supplementary Table 1.

As only data from adults were used for model development by Feng et al., 2008, this model was evaluated as reported, as well as after inclusion of bodyweight-dependent allometric scaling on clearance (fixed exponent: 0.75) and volume of distribution (fixed exponent: 1) of risperidone and 9-OH-risperidone. Therefore, the models evaluated were Model A: Kloosterboer et al., 2021; Model B: Sherwin et al., 2012; Model C: Feng et al., 2008; Model D: Feng et al., 2008 with allometric scaling; and Model E: Thyssen et al., 2010.

### External Evaluation

All the models included in the external evaluation analysis were joint parent-metabolite models, except for the model developed by Thyssen et al., 2010 where only risperidone concentrations were modeled (Supplementary Table 1). As most models were simultaneously predicting risperidone and 9-OH-risperidone’s PK, the plasma concentrations collected and the dose administered were expressed in nmol/mL and nmol, respectively, after dividing by the molecular weight of risperidone (410.485 g/mol) or 9-OH-risperidone (425.91 g/mol).

The additive component of the evaluated error models was expressed in nmol/ml after correcting the reported value in ng/mL with the molecular weight. All the covariates included in the evaluated models were available in our dataset, allowing for a fair evaluation of inter-individual variability (Supplementary Table 1). In the model developed by Thyssen et al., data from various studies were included. Different parameters were estimated for two groups of studies depending on the clinical trial design and sampling scheme. The parameters used for the external evaluation were retrieved for the group of studies that included pediatric patients. In addition, as the model was developed with log-transformed concentrations, the model was also evaluated using log-transformed data. In the model developed by Kloosterboer et al., a different residual error model was used for samples obtained with the dried blood spot technique versus plasma samples. Only the latter error model was used for performing the evaluation as no dried blood spot samples were included in the evaluation dataset. Finally, for the models where a multimodal distribution (mixture model) was assumed for some parameters, the total probability in the population belonging to each subpopulation was fixed, like the rest of the model population parameters, to the value estimated in the respective study. Despite keeping all the parameters fixed, the individual probability of belonging to each subpopulation was estimated for each patient, taking into consideration the respective observations (Carlsson et al., 2009).

The models were implemented using the ADVAN6 subroutine in NONMEM version 7.4 (Icon Development Solutions, Ellicott City, MD, United States). Data manipulation, analysis, and visualization were performed using R (version 4.1.0) and RStudio (version 1.4.1717). The R packages lattice, latticeExtra, and gridExtra were used for preparing the goodness of fit plots (GOF) (Sarkar, 2008; Auguie, 2017; Sarkar and Andrews, 2019).

The external evaluation consisted of two parts. In the first part, the observations (i.e., the concentrations in the external dataset) were compared to the predictions obtained using each model (predictions-based diagnostics). In the second part, 1,000 simulations were performed with each model under evaluation (simulation-based diagnostics). The prediction-corrected visual predictive checks (pcVPCs) were generated by overlaying the observations on the prediction interval of the simulations. In addition, the normalized prediction distribution errors (NPDE) were calculated. The pcVPCs and NPDE are simulation-based diagnostics typically used for the external evaluation of population models (Comets et al., 2008; Bergstrand et al., 2011; Hwang et al., 2017; Nguyen et al., 2017; Cheng et al., 2021).

The observations (OBS) were compared to the population predictions (PRED) to assess the precision and accuracy of the predictions produced by each model. The precision was evaluated using the mean prediction error (PE) and the root mean square error (RMSE) as shown in Eqs. 1, 2. To assess the biases produced by each model, the mean percent error (MPE) and the mean absolute percent error (MAPE) were computed (Equations 3, 4).

$$\mathrm{PE}=\frac{1}{\text{N}}\overset{N}{\underset{i=1}{\sum }}\left(\text{PREDi}-\text{OBSi}\right)\;$$ (1)

$$\mathrm{RMSE}=\sqrt{\frac{1}{\text{N}}\overset{N}{\underset{i=1}{\sum }}{\left(\text{PREDi}-\text{OBSi}\right)}^{2}}$$ (2)

$$\mathrm{MPE}=\frac{100}{\text{N}}\overset{N}{\underset{i=1}{\sum }}\left(\frac{\text{PREDi}-\text{OBSi}}{\text{OBSi}}\right)$$ (3)

$$\mathrm{MAPE}=\frac{100}{\text{N}}\overset{N}{\underset{i=1}{\sum }}\left(\left|\frac{\text{PREDi}-\text{OBSi}}{\text{OBSi}}\right|\right)$$ (4)

pcVPCs were generated using the Perl-speaks-NONMEM tool kit (PsN tool kit; version 3.6.2; Uppsala Pharmacometrics, Uppsala, Sweden) and the R package “xpose4” using 1,000 simulated samples. After retrieving 1,000 simulations using the model under evaluation with NONMEM $SIM subroutine, the NPDE were computed using the R package “npde” (Comets et al., 2008). The NPDE were evaluated statistically (Shapiro–Wilks test for normality, Fisher test for the difference of variance from 1 and t-test for the difference of mean from 0) and visually (histogram of the NPDEs, Q-Q plot, NPDE versus PRED and NPDE versus time) (Comets et al., 2008).

## Results

### Study Sample

A summary of the demographic characteristics of the 62 patients included in the study is presented in Table 1. Among the patients, three had undergone surgery and were on extracorporeal membrane oxygenation (ECMO) support; two were receiving a vasopressor, four hydromorphone, two linezolid, and one metoclopramide. The median (range) number of doses of risperidone recorded per patient during the study was 9 (1–43). The median (range) dose of risperidone administered was 0.250 mg (0.05–2 mg) or 0.017 mg/kg (0.003–0.068). The median (range) daily dose of risperidone administered was 0.450 mg (0.05–6) or 0.025 mg/kg (0.004–0.102).

**TABLE 1:** Population demographics and clinical characteristics of the patients in the external evaluation dataset.

Characteristic	Median (range)
Bodyweight (kg) (*n* = 62)	18.7 (3.64–129)
Post-natal age at first PK draw (years) (*n* = 62)	4.67 (0.16–16.8)
Postmenstrual age at first PK draw (weeks) (*n* = 62)	283 (45–916)
Direct Bilirubin (mg/dl) (*n* = 8)	0.45 (0.0–4.4)
Total Bilirubin (mg/dl) (*n* = 18)	0.40 (0.1–5.6)
Serum Creatinine (mg/dl) (*n* = 48)	0.39 (0.1–0.8)
AST (U/L) (*n* = 19)	35 (12–517)
ALT (U/L) (*n* = 20)	30.5 (12–289)
ALB (g/dl) (*n* = 23)	3.4 (2.3–4.9)
—	Number (%) patients
Sex	—
Male	45 (73)
Female	17 (27)
Age Group	—
Group 1: 31 days ≤ PNA <2 years	23 (37)
Group 2: 2 years ≤ PNA <13 years	29 (47)
Group 3: 13 years ≤ PNA <17 years	10 (16)
RACE	—
White	47 (76)
Black or African American	9 (15)
Unknown or not reported	2 (3)
American Indian or Alaska Native	1 (2)
Native Hawaiian or Pacific Islander	1 (2)
Multiple Races	2 (3)
Obesity status (BMI ≥ 95th percentile) at enrollment	—
Not Obese	17 (27)
Obese (≥95th percentile)	26 (34)
Unknown/Unevaluable^a^	24 (39)
Indication	—
ADHD	4 (6)
Bipolar disorder	3 (5)
Autistic disorder	5 (8)
Behavior disorder	4 (6)
Anxiety	6 (10)
Other^a^	14 (23)
Agitation	10 (16)
Delirium	16 (26)
Route of administration	—
Oral	30 (48)
Nasogastric/Orogastric	9 (15)
Nasojejunal	1 (2)
Transpyloric	7 (11)
Gastrostomy Tube	6 (10)
Jejunostomy Tube	2 (3)
Multiple	7 (11)
Formulation	—
Solution	40 (65)
Tablet	22 (35)

a Other included patients with one of the following conditions or combination of conditions: Angelman’s syndrome, irritability, sleep dysfunction, delirium and agitation, depression, sedation, psychotic episode, anxiety, or depression.

A total of 103 concentrations of risperidone and 112 concentrations of 9-OH-risperidone were quantified and included in the study. The median (range) number of observations per subject was 1 (1–7), both for risperidone and 9-OH-risperidone. A total of 10 concentrations of risperidone and one concentration of 9-OH-risperidone were below the quantification limit (BQL) in the present dataset. However, none of the models externally evaluated reported or modeled the probability of data being BQL using the M3 or M4 Beal methods. Therefore, the BQL data collected in this analysis could not be used, but all the quantifiable concentrations were included.

### External Evaluation

**FIGURE 1:**
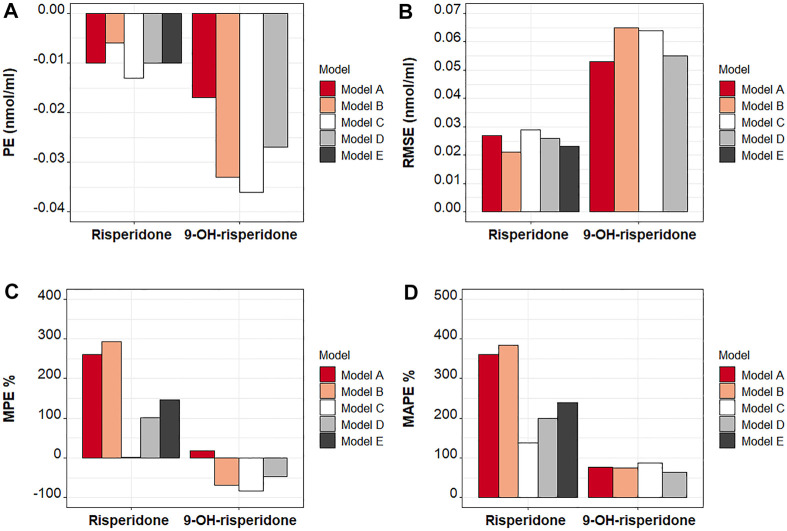
Comparison of quantitative measures of bias and precision among the models included in the external evaluation analysis. Model **(A)**: Kloosterboer et al., 2021; Model **(B)**: Sherwin et al., 2012; Model **(C)**: Feng et al., 2008; Model **(D)**: Feng et al., 2008 with allometric scaling; and Model E: Thyssen et al., 2010. Precision was evaluated using the mean prediction error (PE) and the root mean square error (RMSE). Bias were evaluated using the mean percent error (MPE) and the mean absolute percent error (MAPE).

The predictive performance of the five published models was initially assessed in terms of the precision of the predictions obtained using RMSE and PE **(** Figures 1A,B) and the biases produced using the MAPE and the MPE (Figures 1C,D). The precision of the predictions was similar among the models tested. However, slightly more precise predictions were obtained for risperidone with Model B and secondarily Model D and Model E. For 9-OH-risperidone, Model A and secondarily Model D resulted in more precise predictions. In contrast, there were significant differences among the models in terms of bias. For risperidone, the MPE had a positive value for all the models tested, indicating that the models tended to underestimate the observations **(** Figure 1C). Model C was observed to have a lower bias than the other models, with the MPE% being almost zero. Secondarily, Model D presented less bias than the other models evaluated (Figures 1C,D). For 9-OH-risperidone, the opposite trend was noted, as most of the models tested tended to overestimate the observations, apart from Model A that slightly underestimated the observations (Figure 1C). Considering the MPE and MAPE for 9-OH-risperidone, Models A, B, and D produced similar bias (Figures 1C,D).

**FIGURE 2:**
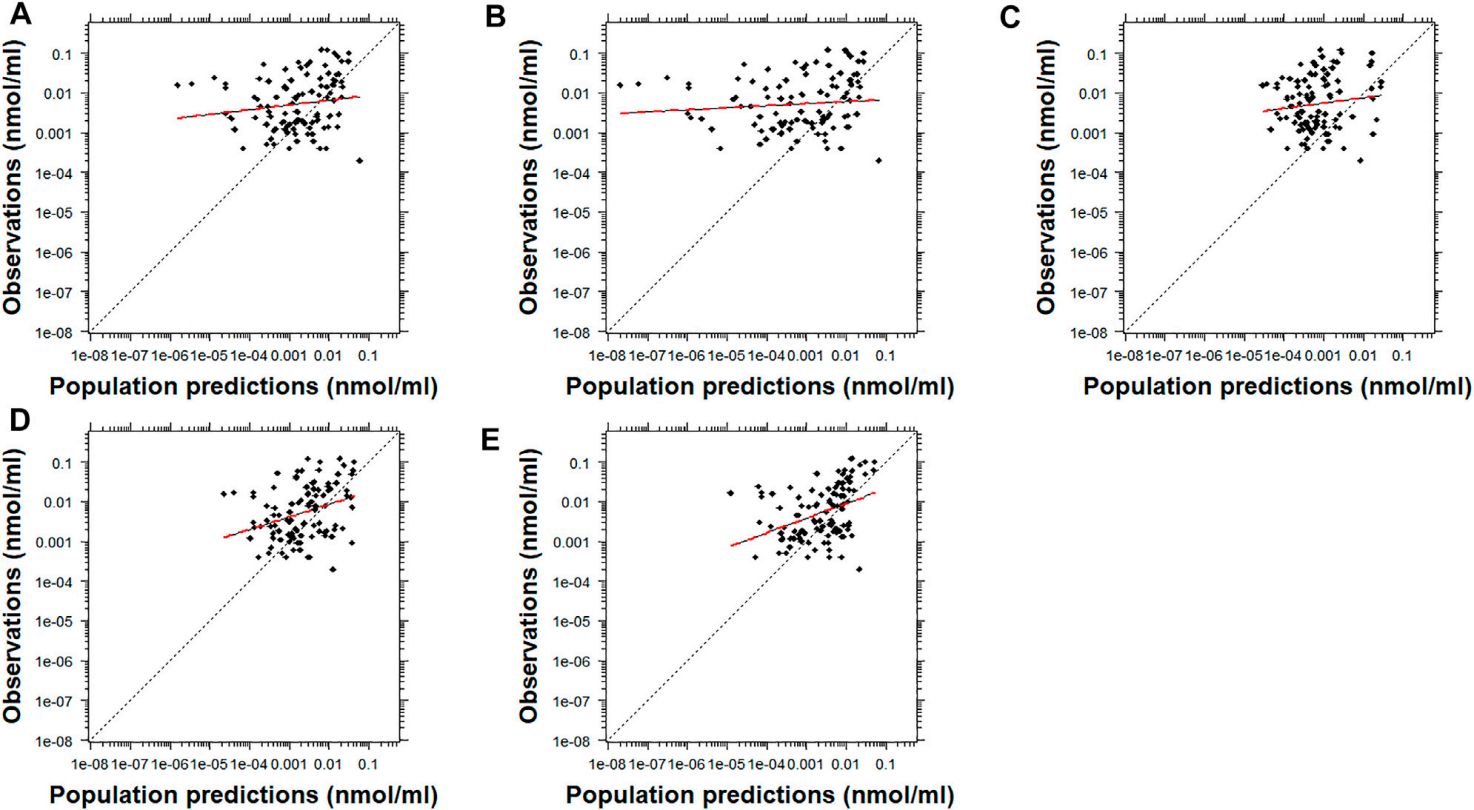
Population predicted concentrations versus observations for risperidone. Model **(A)**: Kloosterboer et al., 2021; Model **(B)**: Sherwin et al., 2012; Model **(C)**: Feng et al., 2008; Model **(D)**: Feng et al., 2008 with allometric scaling; and Model **(E)**: Thyssen et al., 2010. The dashed black and dashed red lines represent the line of identity and the least-squares regression curve, respectively.

**FIGURE 3:**
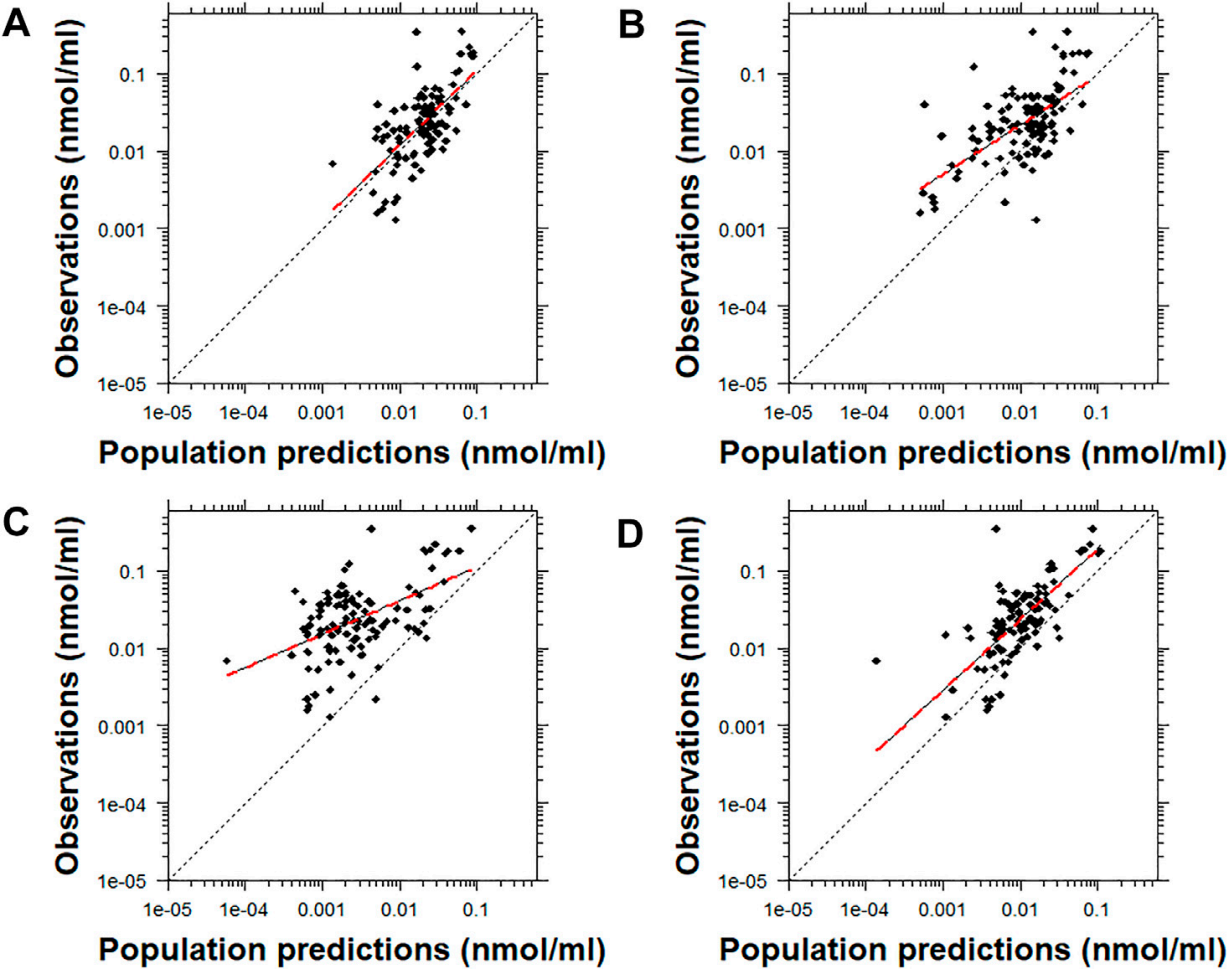
Population predicted concentrations versus observations for the metabolite, 9-OH risperidone. Model **(A)**: Kloosterboer et al., 2021; Model **(B)**: Sherwin et al., 2012; Model **(C)**: Feng et al., 2008; and Model **(D)**: Feng et al., 2008 with allometric scaling. The dashed black and dashed red lines represent the line of identity and the least-squares regression curve, respectively.

After visual inspection of the PRED-versus-OBS plots, it was noted that Model D and Model E resulted in a better performance for risperidone (Figure 2). In comparison, Model A resulted in a better performance for 9-OH-risperidone (Figure 3). Especially for the parent compound, clear trends were noted with all the models under-predicting the observations (Figure 2 **).**

**FIGURE 4:**
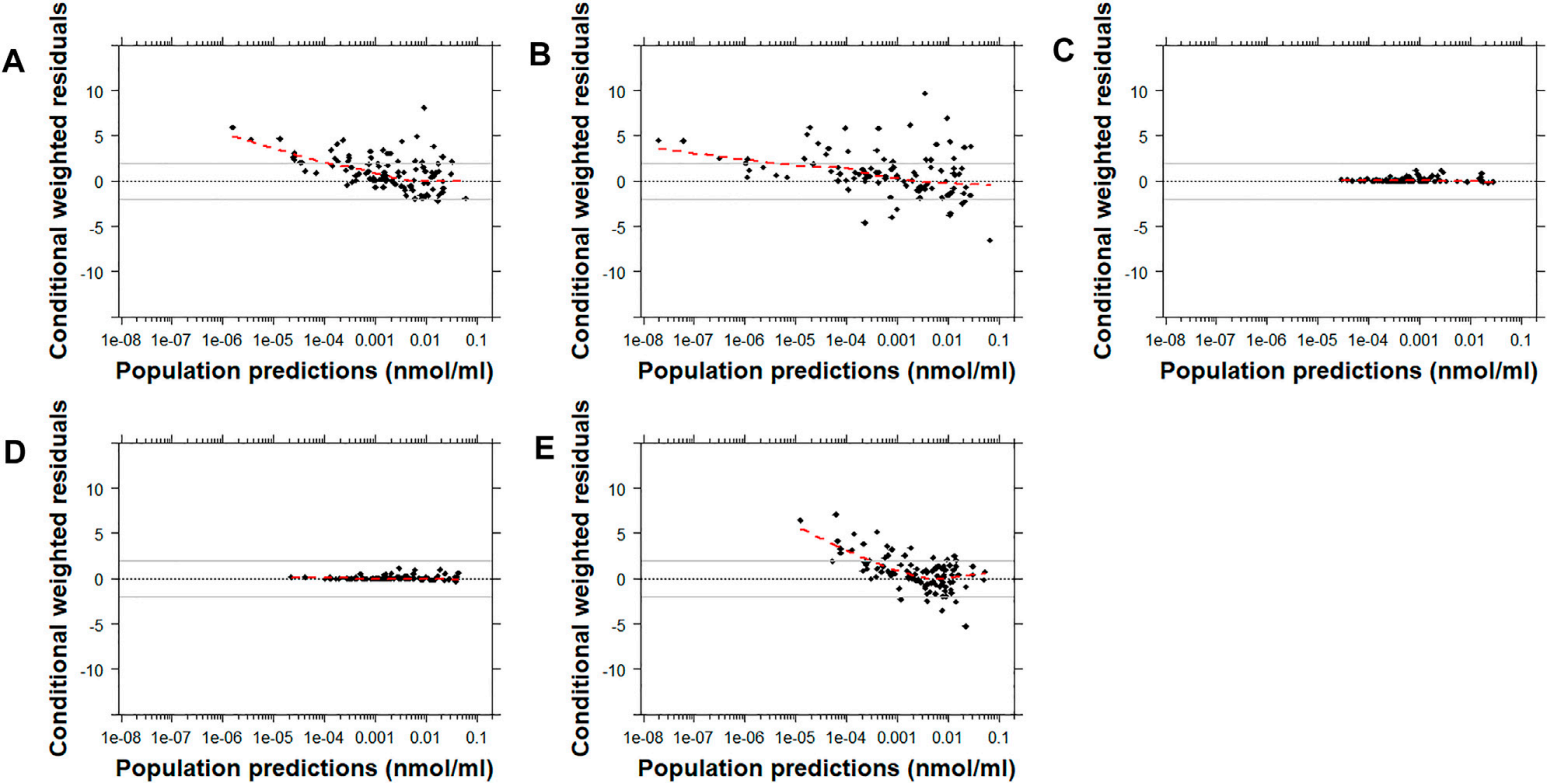
Conditional weighted residuals (CWRES) versus population predictions for risperidone plotted on a log scale. Model **(A)**: Kloosterboer et al., 2021; Model **(B)**: Sherwin et al., 2012; Model **(C)**: Feng et al., 2008; Model **(D)**: Feng et al., 2008 with allometric scaling; and Model **(E)**: Thyssen et al., 2010. The dashed black line corresponds to a CWRES of zero. The solid grey lines correspond to CWRES values of 2 and −2. The dashed red line corresponds to the locally-weighted scatterplot smoothing curve (LOWESS).

**FIGURE 5:**
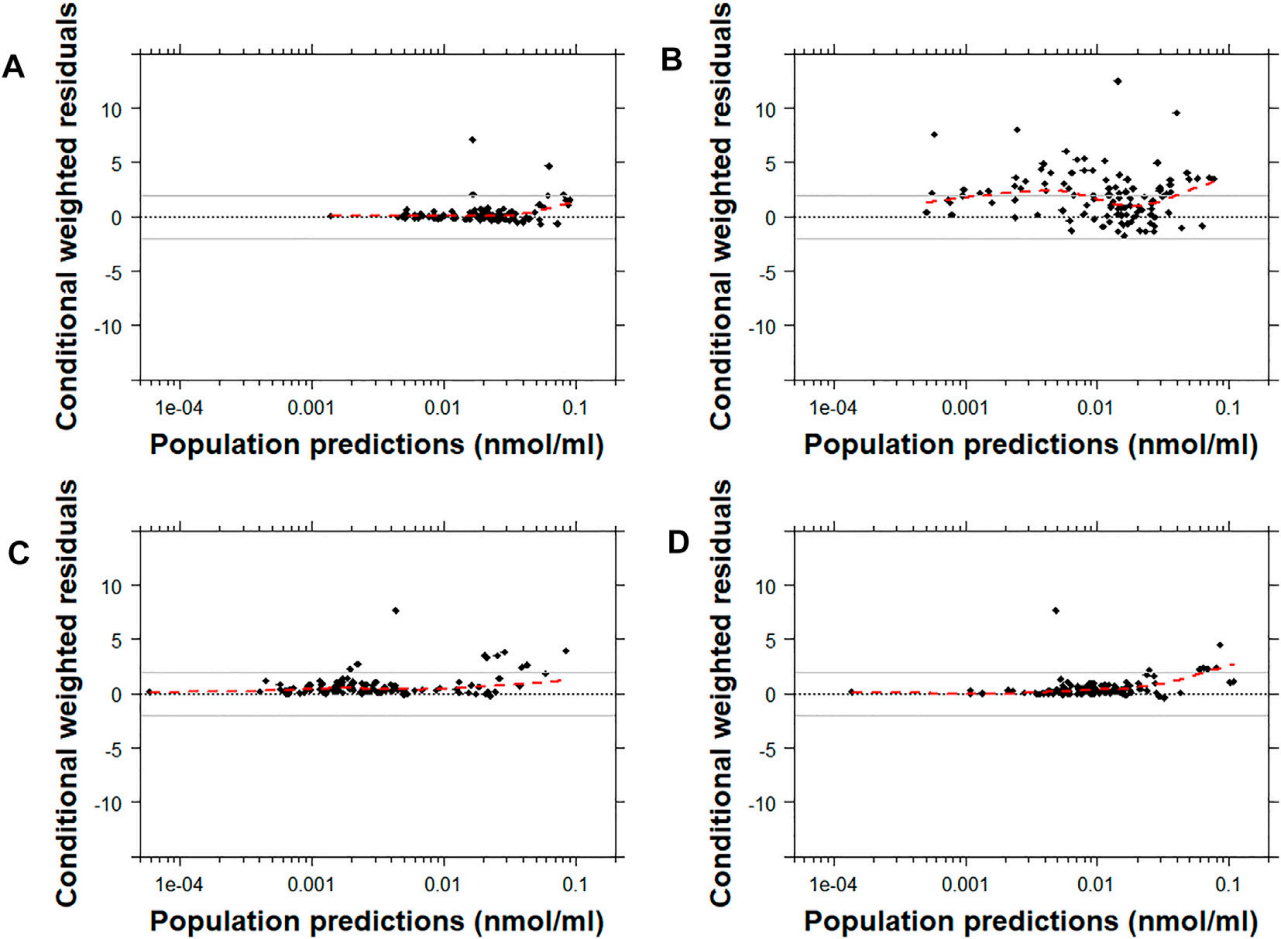
Conditional weighted residuals versus populations predictions for 9-OH risperidone plotted on a log scale. Model **(A)**: Kloosterboer et al., 2021; Model **(B)**: Sherwin et al., 2012; Model **(C)**: Feng et al., 2008; and Model **(D)**: Feng et al., 2008 with allometric scaling. The dashed black line corresponds to a CWRES of zero. The solid grey lines correspond to CWRES values of 2 and -2. The dashed red line corresponds to the locally-weighted scatterplot smoothing curve (LOWESS).

Similarly, the conditional weighted residuals (CWRES)-versus-PRED and CWRES-versus-time after the first dose plots demonstrated that the lower observed concentrations of risperidone were generally under-predicted by most models except for Models C and D (Figure 4 and Supplementary Figures 1, 7). The CWRES-versus-PRED and CWRES-versus-time after the first dose plots generated for 9-OH-risperidone demonstrated that Model A, C, and D performed similarly well, with only a few points deviating. At the same time, Model B resulted in non-normally distributed residuals (Figure 5 and Supplementary Figures 2, 8)**.**

**FIGURE 6:**
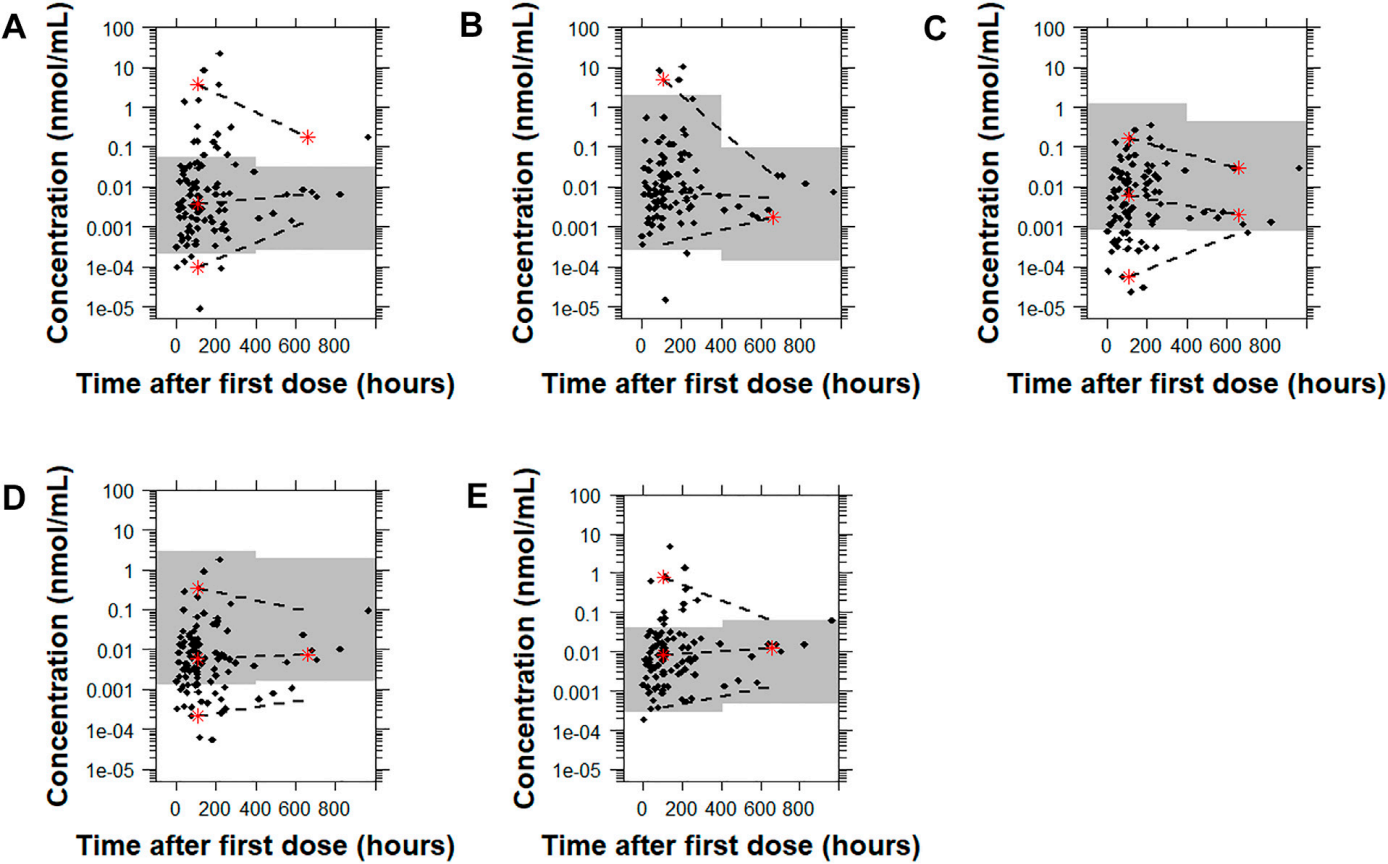
Prediction-corrected visual predictive checks (pcVPCs) of the observed data overlaid on the predictions obtained by performing 1,000 simulations with each risperidone population pharmacokinetic model. Model **(A)**: Kloosterboer et al., 2021; Model **(B)**: Sherwin et al., 2012; Model **(C)**: Feng et al., 2008; Model **(D)**: Feng et al., 2008 with allometric scaling; and Model **(E)**: Thyssen et al., 2010. All pcVPC plots are based on the time after the first dose. The dashed lines represent the 5th, 50th, and 95th percentiles for the observed data, and the gray shaded regions are the 95% prediction interval for the predicted concentrations. The red stars indicate outlying percentiles of the observed data from the prediction interval. The *y* axis is in log-transformed scale. The *x* axis represents the time after first recorded dose. A sample that was collected later than 1,000 h after the first recorded dose was omitted from the graphs to improve visualization. The point was within the prediction interval for all of the models tested except for Model A.

For risperidone, the pcVPC plots showed that all the models had a similar predictive performance (Figure 6 and Supplementary Figure 9). Model B demonstrated the lowest percentage of points outside the 95% prediction interval (4.9% [5 points]) followed by Model E, Model C, Model A, and Model D (11.7% [12 points], 18.4% [19 points], 21.4% [22 points], 26.2% [27 points], respectively). While there was a higher number of points outside the prediction interval with Model C and Model D than Model E or Model B, the distance of the points from the higher or the lower bound of the 95% prediction interval was much lower. Supplementary Figure 3 shows the pcVPCs in a non-log-transformed scale.

**FIGURE 7:**
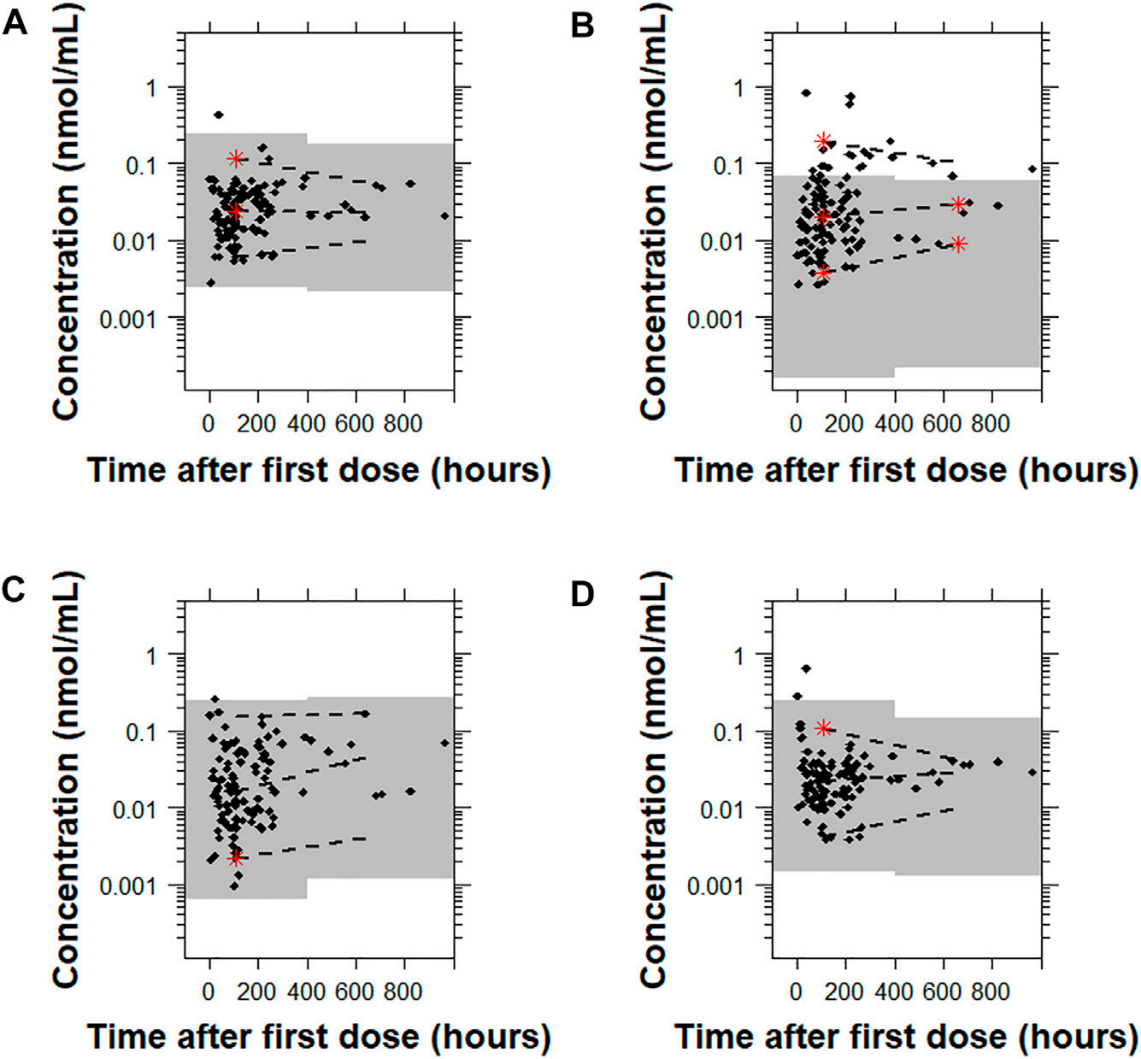
Prediction-corrected visual predictive checks (pcVPCs) where the observed data are overlaid on the predictions obtained by performing 1,000 simulations with each model for 9-OH risperidone. Model **(A)**: Kloosterboer et al., 2021; Model **(B)**: Sherwin et al., 2012; Model **(C)**: Feng et al., 2008; and Model **(D)**: Feng et al., 2008 with allometric scaling. All pcVPC plots are based on the time after the first dose. The dashed lines are the 5th, 50th, and 95th percentiles for the observed data, and the gray shaded regions represent the 95% prediction interval for the predicted concentrations. The red stars indicate outlying percentiles of the observed data from the prediction interval. The *x* axis represents the time after first recorded dose. A sample that was collected later than 1,000 h after the first recorded dose was omitted from the graphs to improve visualization. The point was within the prediction interval for all of the models tested.

For 9-OH-risperidone, the pcVPC plots showed that Models A, C, and D had a similar predictive performance with only 0.9% (1 point), 0.9% (1 point), 1.8% (2 points) of points outside the 95% prediction interval, respectively. In contrast, Model B presented a less adequate predictive performance with 20.5% (23 points) outside the 95% prediction interval (Figure 7 and Supplementary Figure 10).

Hypothesis tests and normality plots performed with the NPDEs generated using 1,000 simulations with each model under evaluation showed that the NPDEs were not normally distributed with a mean of 0 and a variance of 1, with any of the models evaluated (Supplementary Figure 4).

The presence of age-related differences in the models’ misspecification was also explored for both risperidone (Supplementary Figure 5) and 9-OH-risperidone (Supplementary Figure 6). In most cases, greater misspecification was observed in children below 2 years of age and secondarily below 6 years for risperidone. No age-related trend was noted for 9-OH-risperidone.

## Discussion

The predictive performance of five models was evaluated using standard measures of model fitness and goodness-of-fit plots. To our knowledge, this is the first published external evaluation analysis of risperidone and 9-OH-risperidone performed using pediatric data. Despite the high reliability of external evaluation to ensure the predictive capacity of a model (US FDA, 2019; Cheng et al., 2021), this type of evaluation is rarely performed with pediatric data, primarily due to the difficulty of obtaining samples from this vulnerable population. However, this study was made possible as opportunistic data from routine clinical care were collected without burdening the patients with additional blood draws. Given the scarcity of clinical data in infants, children, and adolescents to guide the dosing of risperidone, it is of great importance to assess if the developed models have a good extrapolation to these populations.

The present analysis aimed to externally evaluate population PK models developed in pediatric populations. The only model included that was developed using data only from adults was the model developed by Feng et al., 2008. This model was included because it was developed using the largest number of observations for both the parent and the metabolite. It has never been externally evaluated previously. Also, its structure informed the development of a model with pediatric data (Sherwin et al., 2012). By including the model developed by Feng et al., 2008 in the present analysis, we also aimed to indirectly compare these two models with the same structure and understand if developing the model in children offers a significant advantage compared to developing it in a large number of adults. As a result, a model previously developed in adults by Ji et al., 2016, that had been externally evaluated, was not included in the present analysis.

Many challenges were encountered during the assessment of the results of this analysis due to the inherent variability of risperidone and 9-OH risperidone PK, the significant differences in the models evaluated (Supplementary Table 1), and the populations used for the development of the models and their evaluation. The findings obtained by prediction-based diagnostics and the pcVPC, a simulation-based diagnostic that provides a direct visual comparison between predicted and observed data, generally agreed. Computation of the NPDE, another simulation-based diagnostic, provides information on the accuracy of the predictive performance of a model. However, none of the models tested produced normally distributed NPDE with a mean of zero and variance of one. This probably can be attributed to the general trend of the models to significantly under-predict risperidone (Figures 1, 2) and to over-predict 9-OH-risperidone concentrations (Figures 1, 3). In addition, it should be noted that the NPDE is probably the stringent and most objective diagnostic for model evaluation (Comets et al., 2008; Nguyen et al., 2017).

The model developed by Kloosterboer et al. (Model A) presented the best performance for 9-OH-risperidone, while for risperidone, it presented a relatively modest performance. This model was a 2-compartment model for the parent combined with a 1-compartment for the metabolite, with first-order absorption with lag-time, which did not assume different subpopulations of risperidone clearance. The large variability in risperidone’s PK is mainly attributed to *CYP2D6* genetic polymorphisms affecting its clearance (Sheehan et al., 2010; Kneller et al., 2020; Kneller and Hampel, 2020). Thus, the fact that this source of variability was not accounted for in this model influenced the model’s performance leading to the estimation of population parameters that were less generalizable to other populations. Since the 9-OH-risperidone metabolite is not extensively metabolized and is primarily renally excreted (Vermeir et al., 2008), its primary sources of variability are age and weight (Feng et al., 2008; Kloosterboer et al., 2021). Thus, the excellent predictive performance of Model A for 9-OH-risperidone may be explained by the fact that the model was developed exclusively with data from pediatric patients and included patients with obesity, making it the most similar dataset to the one used for the external evaluation in terms of demographic characteristics of the patients. It should be noted that the dataset used by Sherwin et al., 2012 (Model B) also included exclusively pediatric patients (3–18 years old) and was developed with a similar number of observations. However, the two models had significantly different structures, with Model A requiring much fewer parameters than Model B, potentially contributing to a more accurate estimation of the population parameters, especially those describing the PK of the metabolite.

Three of the models compared [Model B, C and D (Feng et al., 2008 and Sherwin et al., 2012)] had the same structure: a 1-compartment model for the parent and 1-compartment for the metabolite, with first-order absorption, and multimodal risperidone clearance and fraction metabolized (including three subpopulations: poor, intermediate and normal metabolizers). Feng et al. developed a model (Model C [no allometric scaling] and Model D [allometric scaling included]) with data from 490 adult patients (1,236 observations for risperidone and 1,236 for its active metabolite), while Sherwin et al. developed a model (Model B) with data from 41 pediatric patients (163 observations for risperidone and 334 for its active metabolite). The fact that Model B considered the multimodal clearance and was developed using data from a more similar population to the one used for the external evaluation led to the model producing slightly more precise predictions for risperidone (Figures 1A,B and 6). However, the same trend was not present for the metabolite. In addition, overall, based on the other metrics evaluated (MPE%, MAPE%, GOF plots), this model presented significant biases for both the parent and the metabolite (Figures 1C,D, 2 and 3). An explanation for this could be that the model was developed with a relatively small sample size that was also highly heterogeneous (age range: 3–18 years old and weight range: 16–110 kg). In addition, the fraction metabolized for the intermediate metabolizers was fixed to the value of 1 due to estimation difficulties. In contrast, for the normal and the poor metabolizers, the fraction metabolized was estimated at 0.13 and 0.16, respectively. Even though this assumption was also made by Feng et al., possibly due to the availability of a larger number of observations obtained from many patients, a more accurate estimation of the model parameters was made possible. Especially in the case of mixture models, a large sample size is needed to characterize all the subpopulations adequately, and ideally, patients should be monitored for an extended period (Carlsson et al., 2009).

The inclusion of allometric scaling in the model developed by Feng et al., 2008 (Model D) improved the precision of the predictions for risperidone and 9-OH-risperidone while slightly increasing the bias for risperidone but decreasing it for 9-OH-risperidone (Figure 1). In addition, it improved the PRED-versus-OBS plots (Figures 2, 3), as it considered body-weight differences of the pediatric population used for external model evaluation compared to the adult data used for model development. Overall, as Model D showed an adequate performance for risperidone and 9-OH-risperidone, it was considered the model with the best performance for our independent pediatric data set.

The last model [Model E (Thyssen et al.)] evaluated was a 2-compartment model with first-order absorption with a lag time, and multimodal risperidone clearance (including two subpopulations) developed using data from 780 adults and children (3,436 observations for risperidone). Overall, this model showed good predictive performance; however, even though it was developed with the largest number of observations, Models C and D (Feng et al., 2008) slightly outperformed it. This might be because, in the model developed by Thyssen et al., only two subpopulations (poor and normal metabolizers) were considered instead of three (poor, intermediate, and normal metabolizers), which is more reflective of the CYP2D6 phenotypes (Kneller and Hampel, 2020). Also, in the model developed by Feng et al., parent and metabolite data were modeled simultaneously, potentially resulting in a better-informed model compared to the model of Thyssen et al., where only the parent compound was modeled.

This study has several limitations. Given the importance of *CYP2D6* genotype on risperidone PK (PharmGKB, 2021), probably one of the most significant limitations of the study is the fact that *CYP2D6* genotype data were not available in the study dataset. As a result, none of the models evaluated included genotype as a covariate. As *CYP2D6* genotype would account for a large part of the variability noted in risperidone’s clearance and 9-OH-risperidone concentrations, models with a better performance might have been identified. Also, within each model evaluated a different number of subpopulations was assumed. Another limitation was that none of the models considered accounted for the probability of ultrarapid metabolizers (Caudle et al., 2020). Therefore, a different proportion of patients within each subpopulation could have contributed to discrepancies between the model predictions and the observations.

The other limitations of our study were due to the heterogeneity of the opportunistic dataset used for the external evaluation. First, there were notable differences in the demographic and clinical characteristics of the children enrolled in the study (Table 1), including age (0.16–17 years of age) and weight (3.64–129 kg) that are known to exert a significant impact on risperidone’s PK (Aichhorn et al., 2005; Kloosterboer et al., 2021). Based on the ontogeny of CYP2D6, the relative activity of the enzyme is significantly lower in neonates compared to adults (Stevens et al., 2008; van Groen et al., 2021). As a result, the models’ tendency to under-predict parent concentrations might be explained by the fact that 37% of the data included in the dataset used for external evaluation were obtained from patients below 2 years of age. This is also supported by Supplementary Figure 5, which clearly shows the significant impact of maturation on risperidone PK. There is a clear trend of all the models to underpredict the concentration in children below 2 years of age and even below 6 years of age. In contrast, for 9-OH-risperidone, no such trend was noted (Supplementary Figure 6), potentially indicating that its route of elimination is less dependent on maturation.

Despite the known effect of ECMO on the PK of some drugs (Sutiman et al., 2020), the measurements obtained from three patients (5%) on ECMO were included in the analysis. The decision to include these patients in the analysis was made after ensuring that the PEs obtained for these subjects were not different from the average PE estimated for the respective model. Thus, their inclusion was considered a more conservative approach. Similarly, data obtained from a patient receiving metoclopramide concomitantly, a known inhibitor of CYP2D6 (Livezey et al., 2014), were not excluded. Also, different formulations of risperidone were administered through various routes, which could account for some differences noted between the observed data and the models evaluated. Last, different analytical methods were applied to quantify the concentrations of risperidone and 9-OH-risperidone, with different LLOQs, among the studies (Supplementary Table 1).

Despite these shortcomings, this analysis demonstrates the importance of externally evaluating population PK models to assess their generalizability in pediatric populations, especially when these models are intended to guide drug dosing. The external evaluation analyses identified a comparatively better model, while the main factors explaining the high inter-individual variability of risperidone and 9-OH-risperidone were confirmed. As risperidone seems to follow a multimodal clearance, a large amount of data is needed to build a robust and generalizable model and validate it externally. Based on the present analysis results, none of the models evaluated seemed to be generalizable to the population used in this analysis. Thus, a future direction could be establishing a database combining risperidone and 9-OH-risperidone data collected in clinical trials performed so far and during therapeutic drug monitoring. This data could inform the development and evaluation of population PK models designed to guide safe and effective risperidone dosing in the pediatric population.

## The Best Act – Pediatric Trials Network Steering Committee*

**PTN Steering Committee Members:** Daniel K. Benjamin Jr., Christoph Hornik, Kanecia Zimmerman, Phyllis Kennel, and Rose Beci, Duke Clinical Research Institute, Durham, NC; Chi Dang Hornik, Duke University Medical Center, Durham, NC; Gregory L. Kearns, Scottsdale, AZ; Matthew Laughon, University of North Carolina at Chapel Hill, Chapel Hill, NC; Ian M. Paul, Penn State College of Medicine, Hershey, PA; Janice Sullivan, University of Louisville, Louisville, KY; Kelly Wade, Children’s Hospital of Philadelphia, Philadelphia, PA; Paula Delmore, Wichita Medical Research and Education Foundation, Wichita, KS.

**The Eunice Kennedy Shriver National Institute of Child Health and Human Development (NICHD):** Perdita Taylor-Zapata and June Lee.

**The Emmes Company, LLC (Data Coordinating Center):** Ravinder Anand, Gaurav Sharma, Gina Simone, Kim Kaneshige, and Lawrence Taylor.

**PTN Publications Committee:** Chaired by Thomas Green, Ann and Robert H. Lurie Children’s Hospital of Chicago, Chicago, IL.

**The Pediatric Trials Network (PTN) risperidone principal investigators (PIs) are as follows:** Assaf Harofeh Medical Center, Tel Aviv, Israel: Matitiahu Berkovitch (PI)**;** University Hospitals Case Medical Center, Cleveland, OH: David Speicher, (PI)**;** Ann and Robert H. Lurie Childrens Hospital of Chicago, Chicago, IL: William Muller (PI) and Ram Yogev (previous PI)**;** Childrens Hospital of Eastern Ontario, Ottawa, ON: Daniela Pohl (PI), Thierry Lacaze (previous PI), Roger Zemek (previous PI) and Hugh McMullan (previous PI)**;** Children’s Hospital of Wisconsin, Milwaukee, WI: Nathan Thompson (PI) and Beth Drolet (previous PI); Duke University Medical Center Durham, NC: Chi Dang Hornik (PI) and Kevin Watt (previous PI)**;** Alfred I. duPont Hospital for Children/Nemours, Wilmington, DE: Marisa Meyer (PI) and Glenn Stryjeuski (previous PI)**;** Indiana University, Indianapolis, IN: Gregory Sokol (PI), Brenda Poindexter (previous PI) and Scott Denne (previous PI)**;** Oregon Health and Science University, Portland, Oregon: Amira Al-Uzri (PI)**;** University of Arkansas for Medical Sciences, Little Rock, Arkansas.: Laura James (PI)**;** University of Florida—Jacksonville, Jacksonville, FL: Mobeen Rathore (PI)**;** University of Louisville-Kosair Charities Pediatric Clinical Research Unit, Louisville, KY: Janice Sullivan (PI)**;** The University of Maryland Hospital, Baltimore, MD: Caissa Baker-Smith (PI) and Susan Mendley (previous PI)**;** University of North Carolina- Chapel Hill, Chapel Hill, NC: Matt Laughon (PI); and Wesley Medical Center, Wichita, KS: Paula Delmore (previous PI) and Barry Bloom (PI).

## Data Availability

To help expand the knowledge base for pediatric medicine, the Pediatric Trials Network is pleased to share data from its completed and published studies with interested investigators. For requests, please contact: PTN-Program-Manager@dm.duke.edu.
