# Supplementary material for: External Evaluation of Risperidone Population Pharmacokinetic Models Using Opportunistic Pediatric Data
Source: Front Pharmacol. 2022 Mar 17;13:817276. doi: 10.3389/fphar.2022.817276 (PMC8969425; doi:10.3389/fphar.2022.817276)

**Supplementary Material**

**External Evaluation of Risperidone Population Pharmacokinetic Models Using Opportunistic Pediatric Data**

Eleni Karatza^1^, Samit Ganguly^1,2^, Chi D. Hornik^3^, William J. Muller^4^, Amira Al-Uzri^5^, Laura James^6^, Stephen J. Balevic^3^, and Daniel Gonzalez^1^; on behalf of the Best Pharmaceuticals for Children Act – Pediatric Trials Network Steering Committee*

^1^Division of Pharmacotherapy and Experimental Therapeutics, UNC Eshelman School of Pharmacy, The University of North Carolina at Chapel Hill, Chapel Hill, NC, USA; ^2^Regeneron Pharmaceuticals, Inc., Tarrytown, NY, USA; ^3^Duke Clinical Research Institute, Durham, NC, USA; ^4^Ann and Robert H. Lurie Children's Hospital of Chicago, Chicago, IL, USA; ^5^Oregon Health and Science University, Portland, Oregon, USA; and ^6^Arkansas Children's Hospital Research Institute, Little Rock, Arkansas, USA.

^*^See Acknowledgments for listing of committee members.

**Supplementary Table 1.** Description of the studies included in the external evaluation.

| **Reference (Model)** | **Number of Patients** | **Age (years)** | **Weight (kg)** | **Parent observations** | **Metabolite observations** | **Structural model** | **Covariates and error model** | **Analytical method** |
| --- | --- | --- | --- | --- | --- | --- | --- | --- |
| *Kloosterboer et al., 2020  (Model A) | 42 | 6 - 18 | 32.4 (18.3)╪ | 205 | 205 | 2-compartment model for the parent, 1-compartment for the metabolite, and first-order absorption with a lag time | Bodyweight-dependent allometric scaling for clearance (fixed exponent: 0.75) and volume of distribution (fixed exponent: 1)  Additive and proportional residual error models for both the parent and metabolite concentrations. Additional proportional and additive terms were included to account for samples retrieved through the dried blood spot technique. | Ultrahigh performance  liquid chromatography-mass spectrometry (LC-MS/MS);  LLOQ for risperidone: 1 ug/L; and LLOQ for 9-OH- risperidone: 0.7 ug/L |
| Sherwin et al., 2012  (Model B) | 41 | 3 - 18 | 16.8–110 | 163 | 334 | 1-compartment model for the parent, 1-compartment for the metabolite, first-order absorption, and multimodal risperidone clearance and metabolite formation (3 subpopulations) | Bodyweight-dependent allometric scaling for clearance (fixed exponent: 0.75) and volume of distribution (fixed exponent: 1)  Additive and proportional residual error model for both the parent and metabolite concentrations | Liquid chromatography–mass spectrometry assay;  LLOQ for risperidone: 0.2 ng/mL; and  LLOQ for 9-OH- risperidone: 0.2 ng/mL |
| Thyssen et al., 2010  (Model E) | 780 | 6 - 61 | 20–153 | 3436 | N/A | 2-compartment model with first-order absorption and a lag time, and multimodal risperidone clearance and metabolite formation (2 subpopulations) | Bodyweight-dependent allometric scaling for clearance (fixed exponent: 0.75) and volume of distribution (fixed exponent: 1)  Proportional residual error for log-transformed risperidone concentrations | Various validated liquid chromatography-tandem mass spectrometry methods;  LLOQ for risperidone: 0.1-1.0 ng/mL; and  LLOQ for 9-OH- risperidone: 0.1-1.0 ng/mL |
| Feng et al., 2008  (Model C and D with allometric scalling) | 490 | 18 - 93 | 42.7-187.7 | 1236 | 1236 | 1-compartment model for the parent, 1-compartment model for the metabolite, first-order absorption, and multimodal risperidone clearance and metabolite formation (3 subpopulations) | Median age centered using an exponential covariate model on the metabolite's clearance  Additive and proportional residual error models for both the parent and metabolite | Liquid chromatography-mass spectrometry (LC-MS/MS). Detection limit for both compounds: 0.1ng/ml. The Quantification limit was not reported. |

* Study that included children with obesity, ╪median (IQR) while all others are range, NA: not applied, LLOQ: lower limit of quantification

**Supplementary Table 1 continued.** Description of the studies included in the external evaluation.

| **Reference (model)** | **Indications reported** | **Risperidone dosing and formulation information reported** |
| --- | --- | --- |
| Kloosterboer et al., 2020  (Model A) | Autism Spectrum Disorder (ASD) according to the Diagnostic and Statistical Manual of Mental Disorders 4 or 5 and using or starting risperidone for irritability. The majority of children had one or more comorbid psychiatric disorders besides ASD (64.3%), i.e., attention-deficit/hyperactivity disorder (ADHD, 52.4%), oppositional defiant disorder (11.9%), mood disorder (7.1%), post-traumatic stress disorder (4.8%) or anxiety disorder (2.4%). | The median (IQR) risperidone daily dose at the end of follow-up was 1.0 (0.5) mg and 0.02 (0.02) mg/kg. |
| Sherwin et al., 2012  (Model B) | Patients under active treatment with risperidone for a neuropsychiatric disorder. Autistic disorder was the predominant neuropsychiatric diagnosis. | The subjects were treated with risperidone at an average daily dose of 2.0 ± 1.5 mg (0.25–6). |
| Thyssen et al., 2010  (Model E) | 24 children and adolescents (aged 6–17 y) with psychotic and behavior disorders | Flexible dose of 0.25–1.5 or 0.75–1.75 mg/day, twice daily dosing, tablet |
|  | 106 children and adolescents (aged 10–18 y) with bipolar I disorder | Flexible dose of 0.5–2.5 or 3–6 mg/day, tablet |
|  | 261 adolescents (aged 8–17 y) with schizophrenia | Flexible dose of 0.35–0.6 or 3.5–6 mg/day (depending on bodyweight < or >50 kg), oral solution |
|  | 119 adults (aged 18–69 y) with bipolar I disorder | Flexible dose of 1–6 mg/day, tablet |
|  | 140 adults (aged 18–70 y) with bipolar I disorder | Flexible dose of 1–6 mg/day, tablet |
|  | 39 healthy adults (aged 20–55 y) | Single-dose of 2 mg, tablet |
|  | 40 adults (aged 20–61 y) with schizophrenia | Single-dose of 4 mg, tablet |
|  | 36 adults (aged 20–60 y) with schizophrenia or schizoaffective disorder | A single-dose of 4 mg, tablet |
|  | 15 adults with schizophrenia or schizoaffective disorder | Multiple dosing of 15 mg/day, tablet |
| Feng et al., 2008  (Model C and D with allometric scalling) | Adult patients with Alzheimer's disease (AD) and schizophrenia (SZ) | A total of 313 subjects had a once-daily dose of risperidone, and 177 subjects had a twice-daily dose of risperidone with a daily dose range of 0.5-6.0 mg. |

**Supplementary Figure 1.** Conditional weighted residuals (CWRES) versus time for risperidone. Model A: Kloosterboer et al., 2020; Model B: Sherwin et al., 2012; Model C: Feng et al., 2008; Model D: Feng et al., 2008 with allometric scaling; and Model E: Thyssen et al., 2010. The dashed black line corresponds to a CWRES of zero. The solid grey lines correspond to CWRES values of 2 and -2. The dashed red line corresponds to the locally-weighted scatterplot smoothing curve (LOWESS) . The x axis represents the time after first recorded dose. A sample that was collected later than 1000 hours after the first recorded dose was omitted from the graphs to improve visualization. The CWRES for this point were within the range of 2 to -2 with models C, D and E but outside the interval for models A and B.


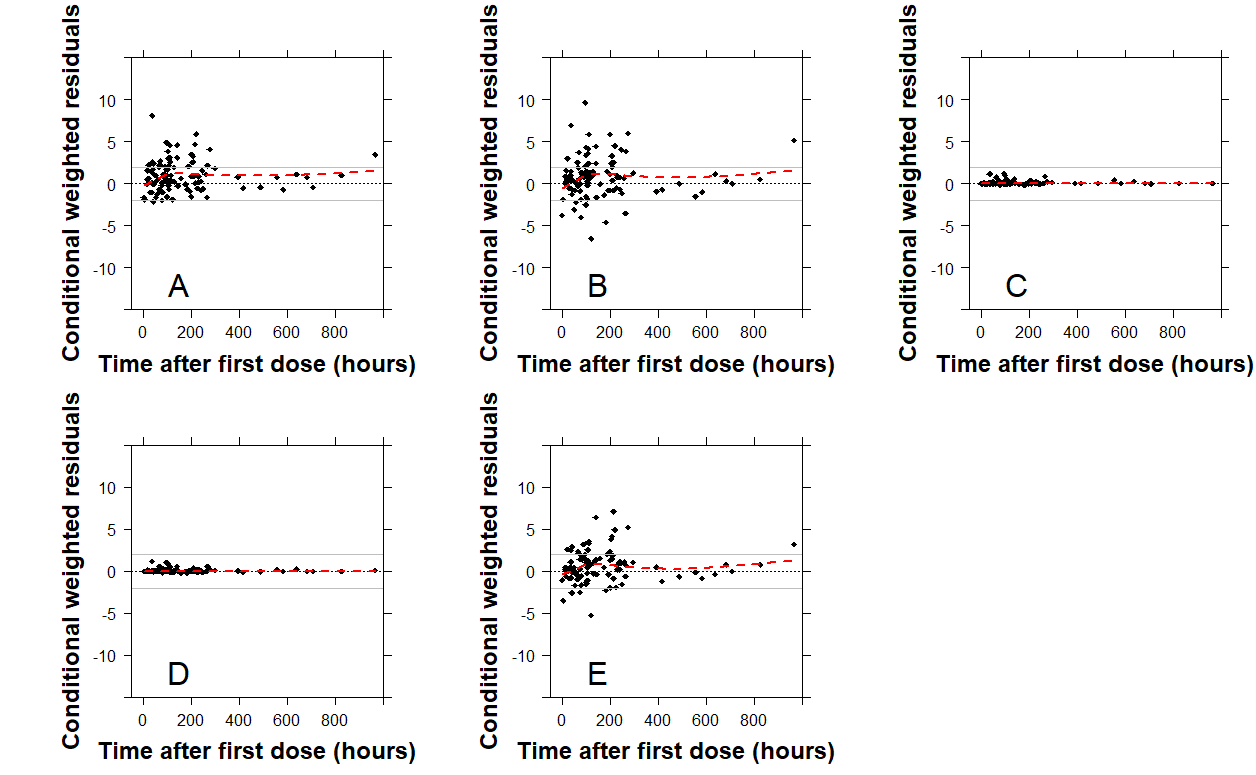


**Supplementary Figure 2.** Conditional weighted residuals versus time for 9-OH-risperidone. Model A: Kloosterboer et al., 2020; Model B: Sherwin et al., 2012; Model C: Feng et al., 2008; and Model D: Feng et al., 2008 with allometric scaling. The dashed black line corresponds to a CWRES of zero. The solid grey lines correspond to CWRES values of 2 and -2. The dashed red line corresponds to the locally-weighted scatterplot smoothing curve (LOWESS). The x axis represents the time after first recorded dose. A sample that was collected later than 1000 hours after the first recorded dose was omitted from the graphs to improve visualization. The CWRES for this point were within the range of 2 to -2 with all the models tested.

**
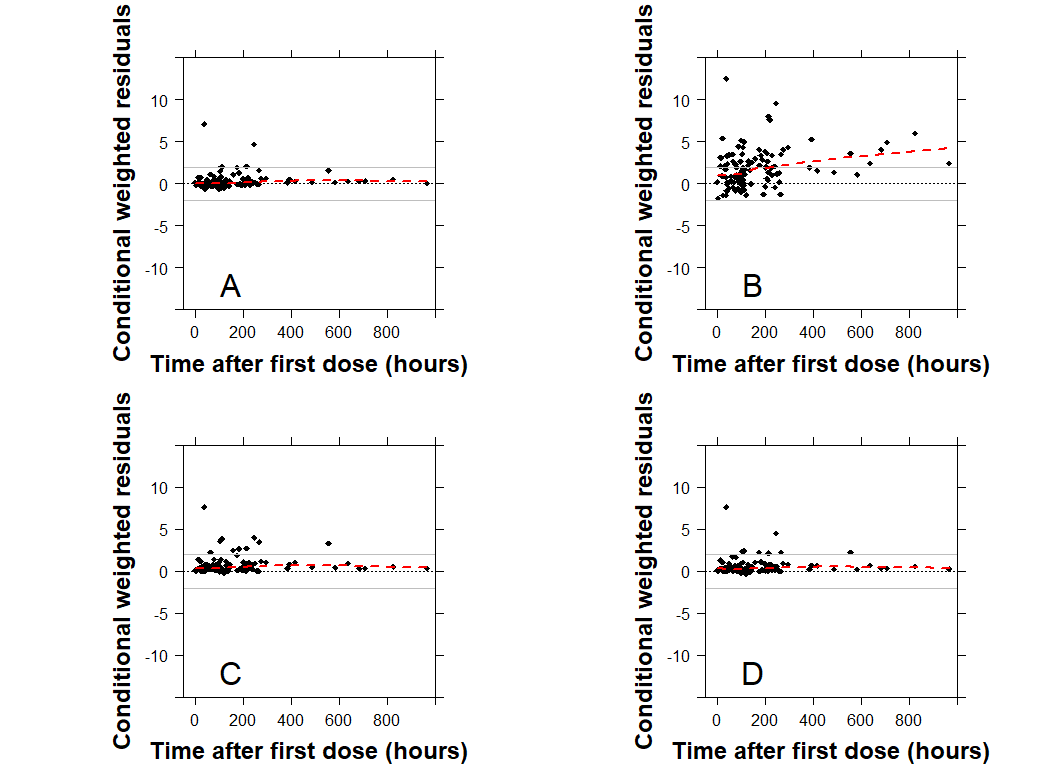
**

**Supplementary Figure 3.** Prediction-corrected visual predictive checks (pcVPCs) of the observed data overlaid on the predictions obtained by performing 1000 simulations with each risperidone population pharmacokinetic model. Model A: Kloosterboer et al., 2020; Model B: Sherwin et al., 2012; Model C: Feng et al., 2008; Model D: Feng et al., 2008 with allometric scaling; and Model E: Thyssen et al., 2010. All pcVPC plots are based on the time after the first dose. The dashed lines represent the 5^th^, 50^th^, and 95^th^ percentiles for the observed data, and the gray shaded regions are the 95% prediction interval for the predicted concentrations. The red stars indicate outlying percentiles of the observed data from the prediction interval. The x axis represents the time after first recorded dose. A sample that was collected later than 1000 hours after the first recorded dose was omitted from the graphs to improve visualization. The point was within the prediction interval for all models tested except for Model A.


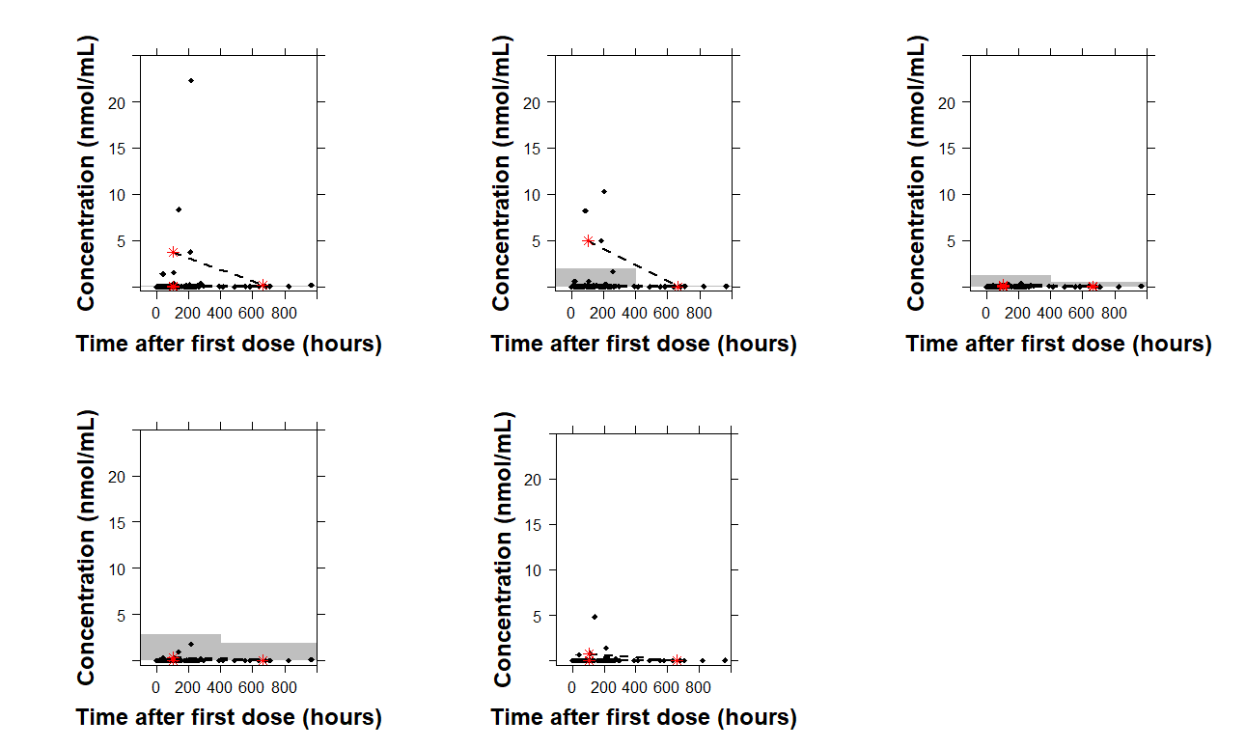


**Supplementary Figure 4.** P-values of the hypothesis tests performed for the NPDE generated using 1000 simulated datasets from each model compared to the pediatric dataset used for external validation were evaluated statistically. The statistical tests performed were the Shapiro–Wilks (SW) test for normality, the Fisher test for the difference of variance from 1, and the t-test to assess whether the mean is different from 0. Model A: Kloosterboer et al., 2020; Model B: Sherwin et al., 2012; Model C: Feng et al., 2008; Model D: Feng et al., 2008 with allometric scaling; and Model E:Thyssen et al., 2010.


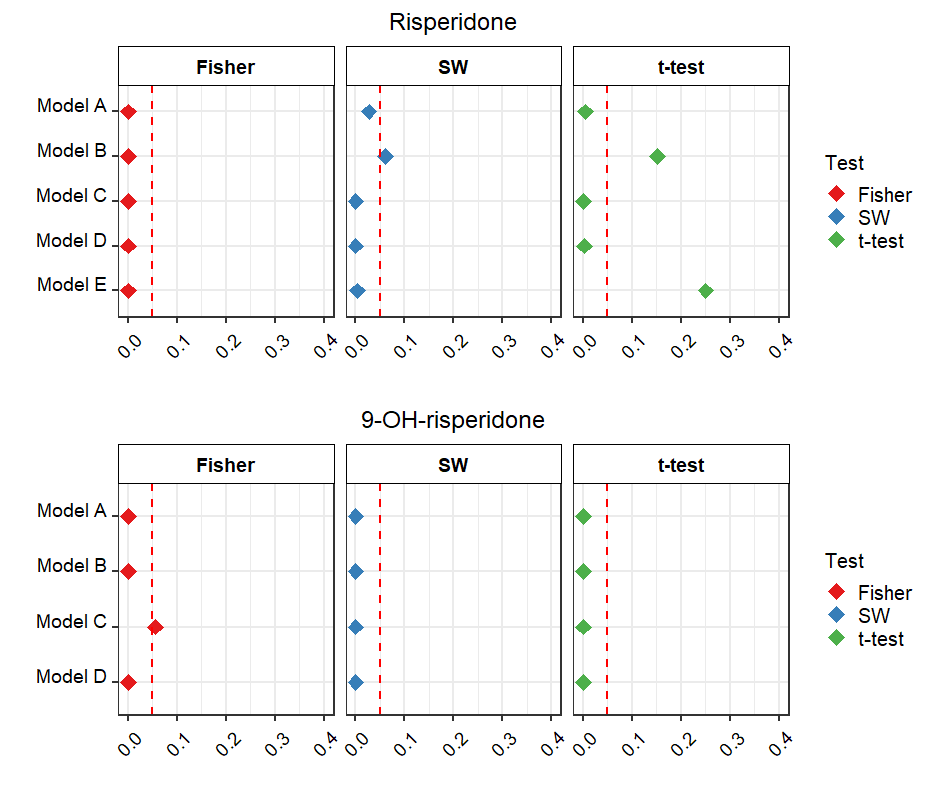


**Supplementary Figure 5.** Population predicted concentrations versus observations for risperidone, stratified by age group. Model A: Kloosterboer et al., 2020; Model B: Sherwin et al., 2012; Model C: Feng et al., 2008; Model D: Feng et al., 2008 with allometric scaling; and Model E: Thyssen et al., 2010. The dashed black and dashed red lines represent the line of identity and the least-squares regression curve, respectively. The black squares represent observations from children 2 years of age and below, the purple circles represent observations from children 2 to 6 years of age, the blue triangles represent observations from children above 6 years up to 12 years of age, and the green diamond represents observations from children above 12 years of age.

**
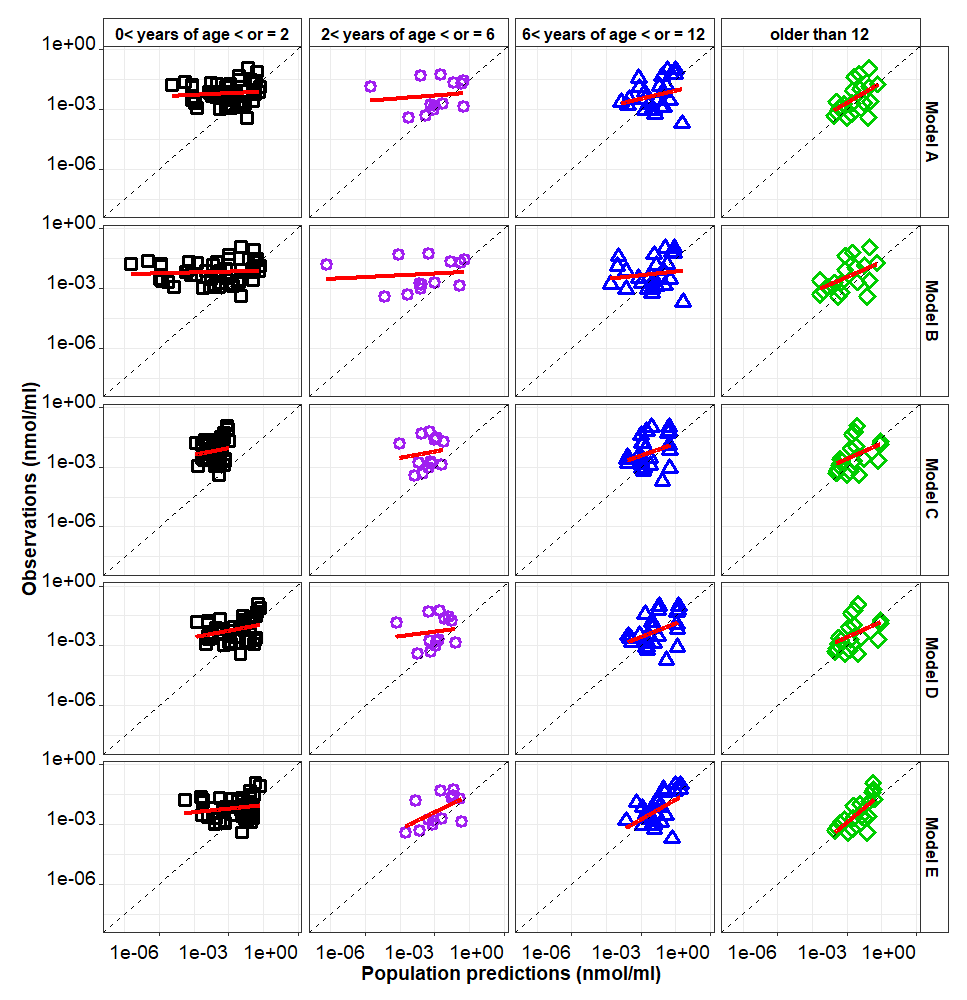
**

**Supplementary Figure 6.** Population predicted concentrations versus observations for the metabolite, 9-OH risperidone, stratified by age group. Model A: Kloosterboer et al., 2020; Model B: Sherwin et al., 2012; Model C: Feng et al., 2008; and Model D: Feng et al., 2008 with allometric scaling. The dashed black and dashed red lines represent the line of identity and the least-squares regression curve, respectively. The black squares represent observations from children 2 years of age and below, the purple circles represent observations from children 2 to 6 years of age, the blue triangles represent observations from children above 6 years up to 12 years of age, and the green diamond represents observations from children above 12 years of age.


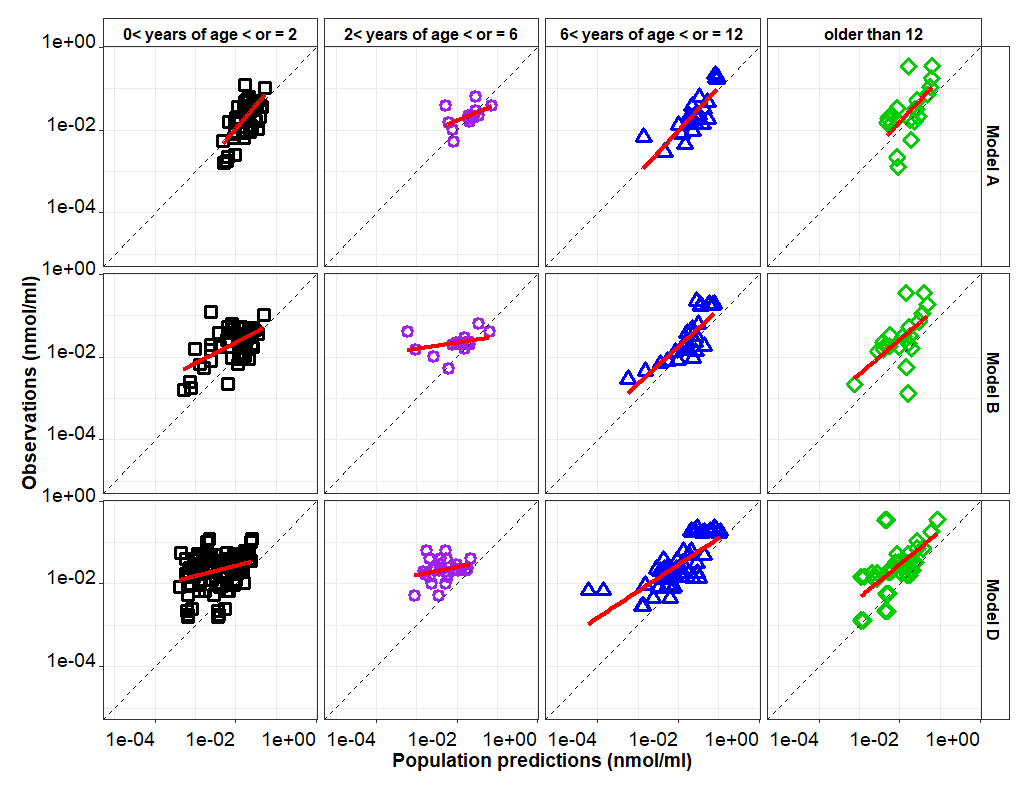


**Supplementary Figure 7**. Conditional weighted residuals (CWRES) versus time for risperidone. Model A: Kloosterboer et al., 2020; Model B: Sherwin et al., 2012; Model C: Feng et al., 2008; Model D: Feng et al., 2008 with allometric scaling; and Model E: Thyssen et al., 2010. The dashed black line corresponds to a CWRES of zero. The solid grey lines correspond to CWRES values of 2 and -2. The dashed red line corresponds to the locally-weighted scatterplot smoothing curve (LOWESS). The x axis represents the time after last dose administered.


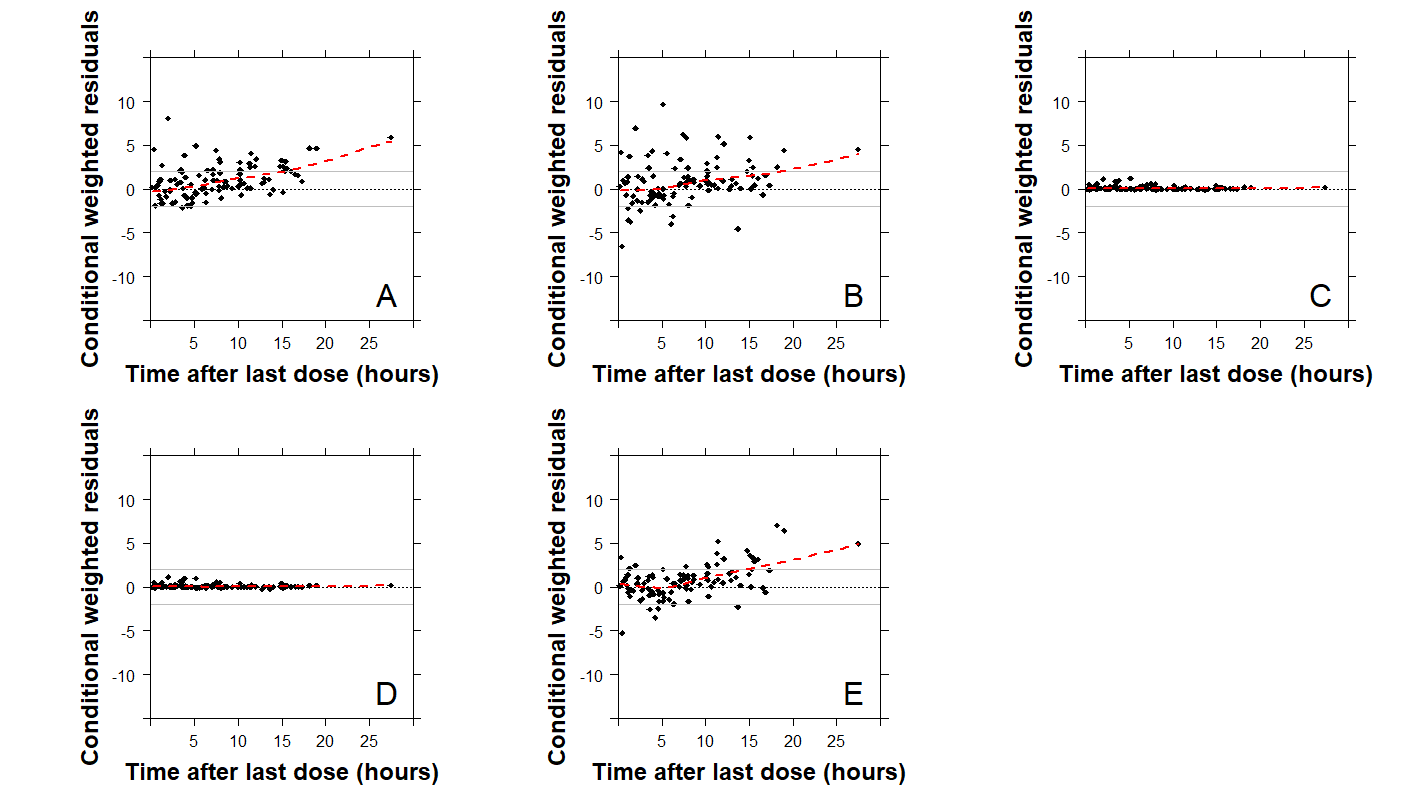


**Supplementary Figure 8.** Conditional weighted residuals versus time for 9-OH-risperidone. Model A: Kloosterboer et al., 2020; Model B: Sherwin et al., 2012; Model C: Feng et al., 2008; and Model D: Feng et al., 2008 with allometric scaling. The dashed black line corresponds to a CWRES of zero. The solid grey lines correspond to CWRES values of 2 and -2. The dashed red line corresponds to the locally-weighted scatterplot smoothing curve (LOWESS). The x axis represents the time after last dose administered.


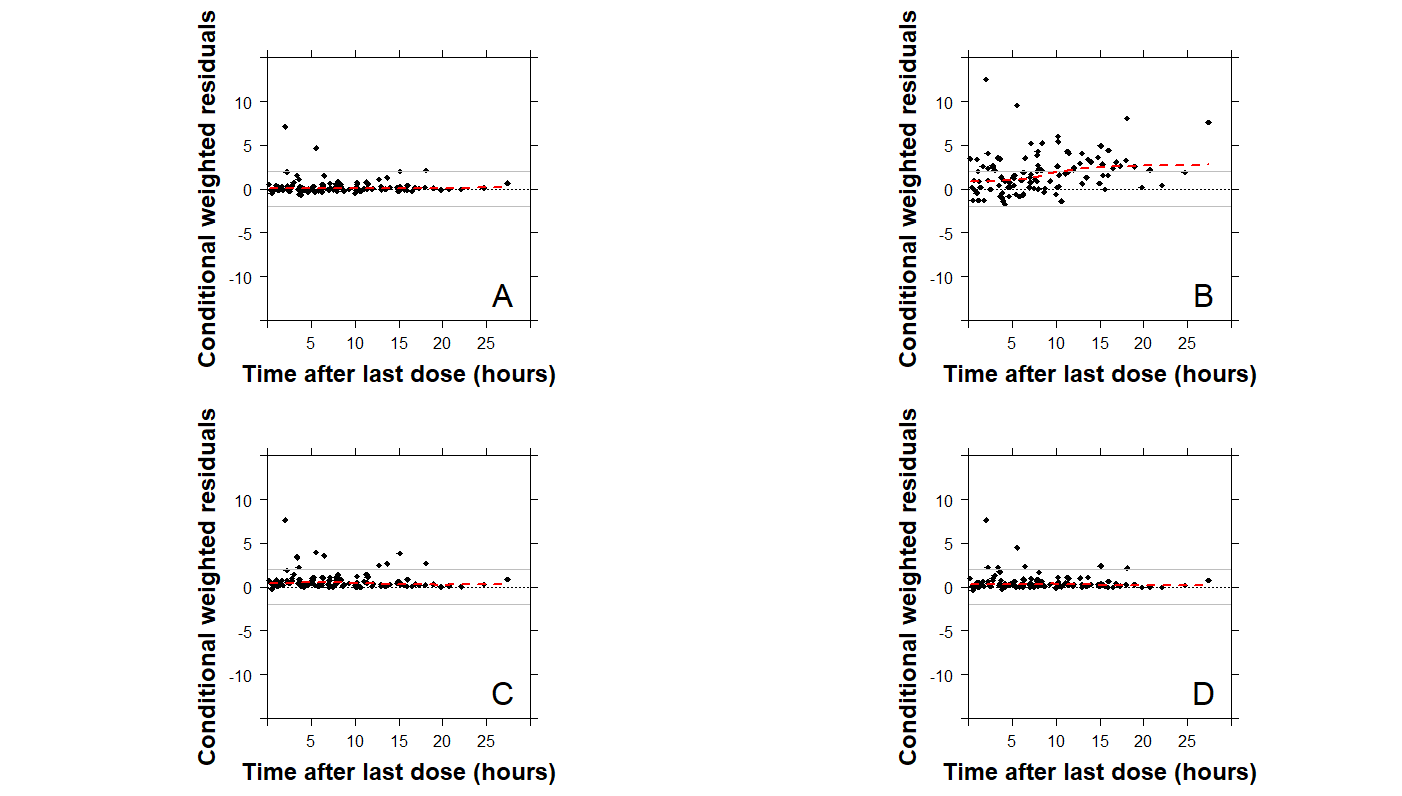


**Supplementary Figure 9.** Prediction-corrected visual predictive checks (pcVPCs) of the observed data overlaid on the predictions obtained by performing 1000 simulations with each risperidone population pharmacokinetic model. Model A: Kloosterboer et al., 2020; Model B: Sherwin et al., 2012; Model C: Feng et al., 2008; Model D: Feng et al., 2008 with allometric scaling; and Model E: Thyssen et al., 2010. All pcVPC plots are based on the time after the first dose. The dashed lines represent the 5^th^, 50^th^, and 95^th^ percentiles for the observed data, and the gray shaded regions are the 95% prediction interval for the predicted concentrations. The red stars indicate outlying percentiles of the observed data from the prediction interval. The y axis is in log-transformed scale. The x axis represents the time after last dose administered.


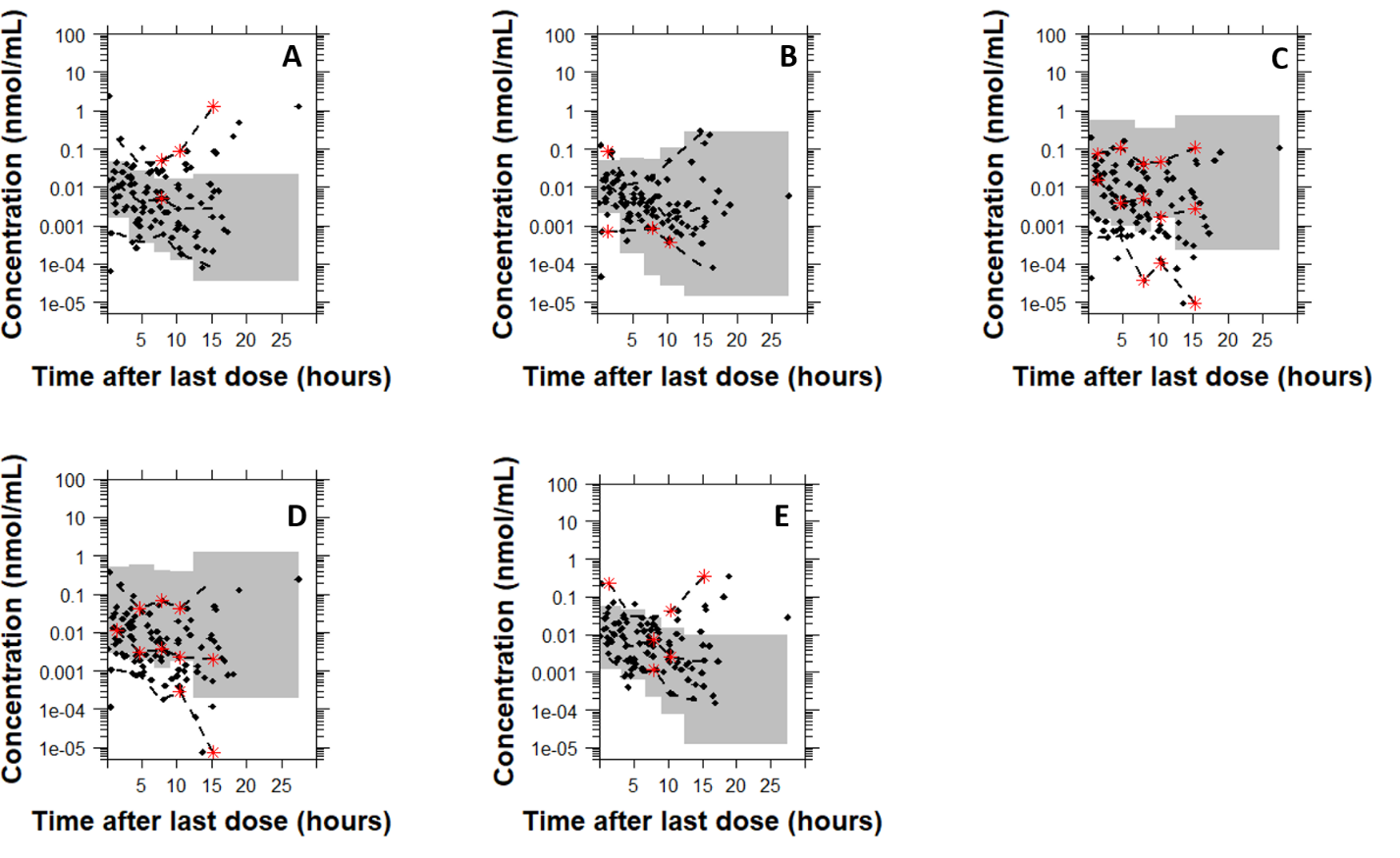


**Supplementary Figure 10.** Prediction-corrected visual predictive checks (pcVPCs) of the observed data overlaid on the predictions obtained by performing 1000 simulations with each 9-OH-risperidone population pharmacokinetic model. Model A: Kloosterboer et al., 2020; Model B: Sherwin et al., 2012; Model C: Feng et al., 2008; Model D: Feng et al., 2008 with allometric scaling; and Model E: Thyssen et al., 2010. All pcVPC plots are based on the time after the first dose. The dashed lines represent the 5^th^, 50^th^, and 95^th^ percentiles for the observed data, and the gray shaded regions are the 95% prediction interval for the predicted concentrations. The red stars indicate outlying percentiles of the observed data from the prediction interval. The y axis is in log-transformed scale. The x axis represents the time after last dose administered.


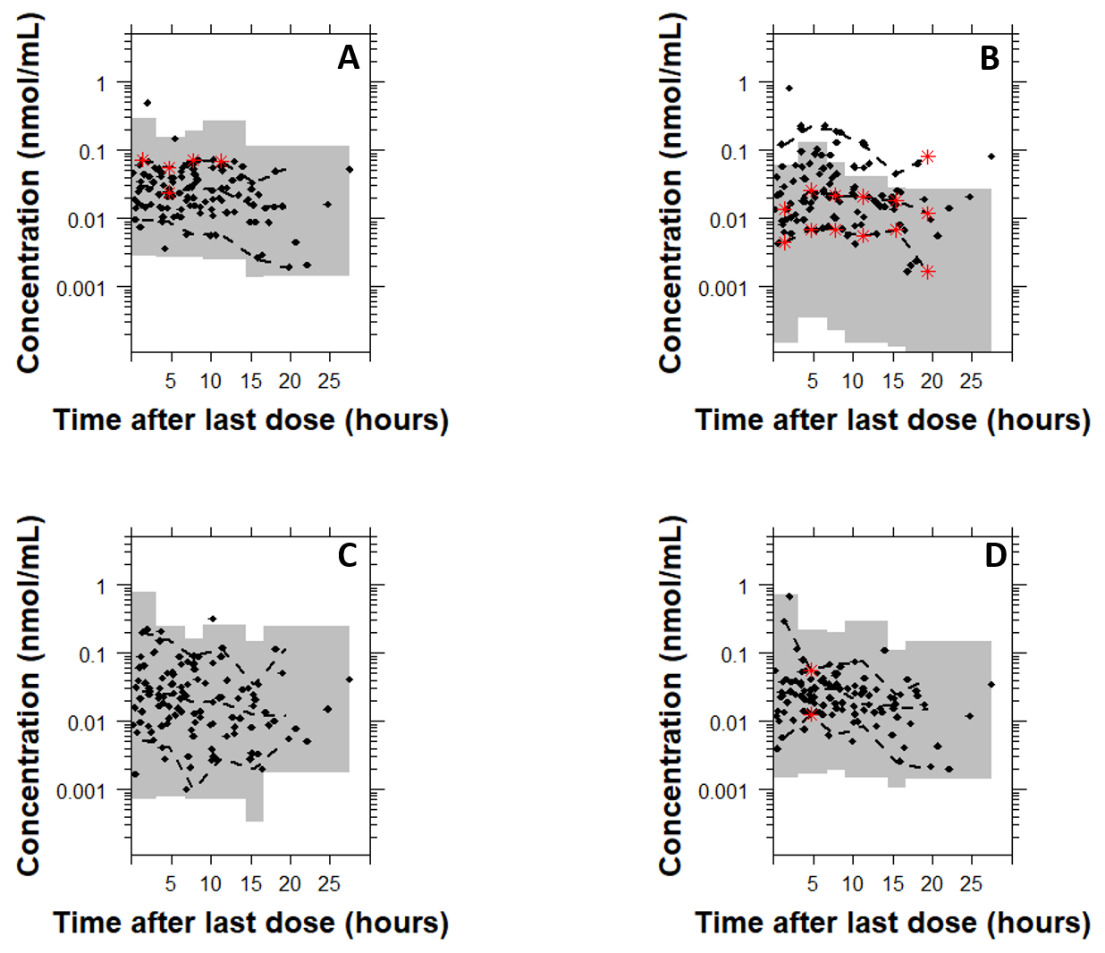

Supplement: Supplementary file 1 [file DataSheet1.docx]
